# Interventions to improve outcomes for caregivers of patients with advanced cancer: a meta-analysis

**DOI:** 10.1093/jnci/djad075

**Published:** 2023-06-05

**Authors:** Ronald Chow, Jean J Mathews, Emily YiQin Cheng, Samantha Lo, Joanne Wong, Sorayya Alam, Breffni Hannon, Gary Rodin, Rinat Nissim, Sarah Hales, Dio Kavalieratos, Kieran L Quinn, George Tomlinson, Camilla Zimmermann

**Affiliations:** Department of Supportive Care, Princess Margaret Cancer Centre, University Health Network, Toronto, ON, Canada; Temerty Faculty of Medicine, University of Toronto, Toronto, ON, Canada; Division of Palliative Medicine, Department of Medicine and Department of Oncology, Queen’s University, Kingston, ON, Canada; Department of Supportive Care, Princess Margaret Cancer Centre, University Health Network, Toronto, ON, Canada; Temerty Faculty of Medicine, University of Toronto, Toronto, ON, Canada; Department of Supportive Care, Princess Margaret Cancer Centre, University Health Network, Toronto, ON, Canada; Department of Supportive Care, Princess Margaret Cancer Centre, University Health Network, Toronto, ON, Canada; Palliative Medicine, Sobell House, Oxford University Hospitals NHS Foundation Trust, Oxford, UK; Department of Supportive Care, Princess Margaret Cancer Centre, University Health Network, Toronto, ON, Canada; Temerty Faculty of Medicine, University of Toronto, Toronto, ON, Canada; Department of Medicine, University Health Network, Toronto, ON, Canada; Department of Supportive Care, Princess Margaret Cancer Centre, University Health Network, Toronto, ON, Canada; Temerty Faculty of Medicine, University of Toronto, Toronto, ON, Canada; Dalla Lana School of Public Health, University of Toronto, Toronto, ON, Canada; Centre for Mental Health, University Health Network, Toronto, ON, Canada; Department of Supportive Care, Princess Margaret Cancer Centre, University Health Network, Toronto, ON, Canada; Temerty Faculty of Medicine, University of Toronto, Toronto, ON, Canada; Centre for Mental Health, University Health Network, Toronto, ON, Canada; Department of Supportive Care, Princess Margaret Cancer Centre, University Health Network, Toronto, ON, Canada; Temerty Faculty of Medicine, University of Toronto, Toronto, ON, Canada; Centre for Mental Health, University Health Network, Toronto, ON, Canada; Division of Palliative Medicine, Department of Family and Preventive Medicine, Emory University, Atlanta, GA, USA; Temerty Faculty of Medicine, University of Toronto, Toronto, ON, Canada; Dalla Lana School of Public Health, University of Toronto, Toronto, ON, Canada; Division of General Internal Medicine and Palliative Care, Department of Medicine, Sinai Health System, Temmy Latner Centre for Palliative Care, Toronto, ON, Canada; Temerty Faculty of Medicine, University of Toronto, Toronto, ON, Canada; Department of Medicine, University Health Network, Toronto, ON, Canada; Dalla Lana School of Public Health, University of Toronto, Toronto, ON, Canada; Department of Supportive Care, Princess Margaret Cancer Centre, University Health Network, Toronto, ON, Canada; Temerty Faculty of Medicine, University of Toronto, Toronto, ON, Canada; Department of Medicine, University Health Network, Toronto, ON, Canada; Dalla Lana School of Public Health, University of Toronto, Toronto, ON, Canada

## Abstract

**Background:**

Family caregivers of patients with advanced cancer often have poor quality of life (QOL) and mental health. We examined the effectiveness of interventions offering support for caregivers of patients with advanced cancer on caregiver QOL and mental health outcomes.

**Methods:**

We searched Ovid MEDLINE, EMBASE, Cochrane CENTRAL, and Cumulative Index to Nursing and Allied Health Literature databases from inception through June 2021. Eligible studies reported on randomized controlled trials for adult caregivers of adult patients with advanced cancer. Meta-analysis was conducted for primary outcomes of QOL, physical well-being, mental well-being, anxiety, and depression, from baseline to follow-up of 1-3 months; secondary endpoints were these outcomes at 4-6 months and additional caregiver burden, self-efficacy, family functioning, and bereavement outcomes. Random effects models were used to generate summary standardized mean differences (SMD).

**Results:**

Of 12 193 references identified, 56 articles reporting on 49 trials involving 8554 caregivers were eligible for analysis; 16 (33%) targeted caregivers, 19 (39%) patient–caregiver dyads, and 14 (29%) patients and their families. At 1- to 3-month follow-up, interventions had a statistically significant effect on overall QOL (SMD = 0.24, 95% confidence interval [CI] = 0.10 to 0.39); *I*^2^ = 52.0%), mental well-being (SMD = 0.14, 95% CI = 0.02 to 0.25; *I*^2^ = 0.0%), anxiety (SMD = 0.27, 95% CI = 0.06 to 0.49; *I*^2^ = 74.0%), and depression (SMD = 0.34, 95% CI = 0.16 to 0.52; *I*^2^ = 64.4) compared with standard care. In narrative synthesis, interventions demonstrated improvements in caregiver self-efficacy and grief.

**Conclusions:**

Interventions targeting caregivers, dyads, or patients and families led to improvements in caregiver QOL and mental health. These data support the routine provision of interventions to improve well-being in caregivers of patients with advanced cancer.

Family caregivers are relatives or friends who provide unpaid care for patients, assisting with physically, emotionally, and socially demanding tasks (1-3). More than 1 in 10 adults in the United States and Europe are family caregivers, with cancer among the most common diagnoses of care recipients (4,5). Caregivers of patients with advanced cancer (defined in this paper as stage III or greater) are particularly vulnerable compared with those caring for patients with earlier stage cancers, as patients’ increased symptom burden, decreased functional status, and the need for advance care planning and end-of-life discussions lead to increased caregiver demands (1). These caregivers often experience poor quality of life (QOL), a multidimensional construct encompassing physical, emotional, social, financial, and spiritual aspects of well-being (6). In particular, caregivers of patients with advanced cancer may have poor mental health, including depression and anxiety (7-12), as well as physical symptoms including fatigue, sleep disturbance, loss of appetite, and pain (13,14). The tendency of modern medicine to prioritize patient-centered aspects of care including autonomy and confidentiality has led to an unintended neglect of their caregivers, particularly in the advanced cancer setting (15).

Interventions to support caregivers of patients with advanced cancer may be categorized according to their target population of individual caregivers, caregiver-patient dyads, or patients and their families (16-18). Two comprehensive meta-analyses have examined caregiver interventions (19,20)—one in caregivers of patients with cancer mainly at earlier stages (19) and the other in caregivers of patients in the terminal phase of any disease (20)—both were published more than a decade ago. Other meta-analyses were limited to specific psychosocial interventions (21,22) or to interventions in home settings (23) or only assessed impact on caregivers of interventions directed at patients (24-28). Given the heavy burden of caregiving and high levels of distress in caregivers of patients with advanced cancer (15), the objective of the current review was to determine the effect of interventions offering support for caregivers of patients with advanced cancer on caregiver QOL and mental health outcomes.

## Methods

The protocol for this systematic review and meta-analysis was registered with the International Prospective Register of Systematic Reviews (PROSPERO CRD42019136321). This review was conducted and reported in accordance with the Cochrane Handbook for Systematic Reviews (29) and the Preferred Reporting Items for Systematic reviews and Meta-Analyses statement (30).

### Identification and selection of studies

With assistance from a health science librarian, we searched the following databases from inception through June 2021: Ovid MEDLINE, EMBASE, Cochrane CENTRAL Register of Controlled Trials, and Cumulative Index to Nursing and Allied Health Literature (CINAHL). MeSH subject headings and specific search terms were used to execute the search (Supplementary Methods 1, available online), which was restricted to clinical trials and English-language publications. Two reviewers (RC and CZ) screened references from retrieved papers and previous systematic reviews to retrieve additional studies not identified by the search strategy. Two of 4 reviewers (RC, SA, EYC, JW) independently evaluated all studies for eligibility using predefined eligibility criteria; discrepancies were resolved by discussion and, if necessary, with the input of a further reviewer (CZ).

### Study eligibility criteria

We included studies that reported on randomized controlled trials of interventions for adult (aged 18 years and older) caregivers of adult patients with advanced (stage III or IV) cancer; to reduce heterogeneity, only trials in which all patients had advanced cancer were included. Interventions needed to be either psychoeducational, skills training, counseling, or team-based interventions offering direct or indirect support with caregiving or coping. The interventions could be directed at the caregiver, the patient–caregiver dyad, or the patient and/or his or her family, provided that caregiver outcomes were measured. Interventions specifically designed to target patient–caregiver dyads were classified as dyad interventions, whereas those targeting the patient alone, or targeting the patient and 1 or more family members who were not specified as being caregivers, were classified as directed at the patient and/or his or her family. Studies that assessed complementary therapies (eg, massage) were excluded because they did not meet the definition of a psychoeducational, skills training, counseling, or team-based intervention, and those that assessed interventions targeting only 1 symptom (eg, sleep) were excluded because these interventions were tailored to focus only on that particular symptom rather than on improving overall QOL and mental health. Comparators could be usual care or an active control (Supplementary Methods 2, available online). Studies published only as abstracts were excluded because abstracts often consist of partial or interim data that may change with publication of the final study, and quality of reporting is often poor (31,32). Studies with sample size less than 20 per trial arm were excluded because of greater risk of publication bias and lower trial quality associated with smaller samples (33).

### Data extraction and risk of bias assessment

Two of 4 reviewers (RC, JJM, SL, EYC) independently extracted data from the included studies using a standardized, prepiloted data extraction form. The target of the intervention was classified as being the caregiver, the patient–caregiver dyad, or the patient and his or her family (15). Disagreements were resolved by discussion, with input from a further reviewer (CZ), if necessary. Missing data for meta-analysis were requested from study authors up to 2 times; if no response was received after the second request, the study was not included in the meta-analysis. The same reviewers used the Cochrane Risk of Bias Tool 2.0 (34) to assess the risk of bias of the included trials, with disagreements resolved in the same manner. The tool was used to assign each trial a rating of low, high, or some concerns of bias using a standardized method (Supplementary Methods 3, available online) (35). For cluster-randomized trials, the modified Cochrane Risk of Bias Tool 2.0 for cluster-randomized trials was used (36). Publication bias was assessed using funnel plots and Egger tests for all primary outcomes at 1-3 months.

### Synthesis and statistical analysis

A narrative synthesis was conducted to describe data for all outcomes reported for each trial. Primary outcomes of overall QOL, QOL subscales of physical and mental well-being, depression, and anxiety were selected a priori for meta-analysis. All of these outcomes were predefined as occurring while the patient was living, to avoid introduction of confounding and heterogeneity due to the impact of the patient’s death on caregivers’ QOL and mental health. Secondary outcomes were described only in the narrative synthesis and comprised caregiver burden (including outcomes of caregiver burden, stress, or strain), caregiver self-efficacy (including self-efficacy, competence, mastery, knowledge, or preparedness), family functioning (including family relationships and family functioning), and bereavement outcomes (including grief and depression after death of the patient).

A meta-analysis was conducted for all primary outcomes; study data were extracted separately by time from baseline to 1-3 months (primary endpoint) and 4-6 months (secondary endpoint) follow-up; these endpoints were chosen because they are commonly used in trials of caregiver interventions and differentiate between short- and longer-term effects (24). Similar to a previous review (24), if a study reported outcomes more than once within the same 1- to 3-month or 4- to 6-month interval, the last time point was used; outcomes reported between these 2 intervals were categorized with the 1- to 3-month interval. For studies with multiple measures assessing the same outcome (eg, 2 measures for QOL), we established a hierarchy for inclusion in the meta-analysis, based on authors’ designation of the measure as the primary outcome, number of items (full measures preferred over abbreviated ones), and validation for use in caregivers. If a study included a brief and more extensive intervention, we conservatively used the brief intervention for the main analysis and conducted a sensitivity analysis using the extensive intervention.

Because measures to evaluate each outcome varied among trials, summary statistics were reported as standardized mean differences (SMDs) for each trial, corrected for scale directionality when necessary, and calculated using a Hedges adjusted G estimator to correct for small sample bias (37). SMDs of 0.2, 0.5, and 0.8 represent small, moderate, and large effects, respectively (38). To account for statistical heterogeneity of treatment effects across trials, random effects (Dersimonian and Laird) models were used to generate summary SMDs. The Hartung-Knapp adjustment was used for confidence intervals and statistical tests (39). The proportion of the total between-study variance in the treatment effects attributable to between-study heterogeneity (and not sampling variability) was documented using the *I*^2^ statistic (40). Heterogeneity was also assessed using the between-study variance of the treatment effect ($\tau^{2}$) and the Cochrane *Q* statistic. Subgroup analyses were conducted to compare pooled results of trials by intervention and by risk of bias. StataBE 17.0 (StataCorp) was used for all analyses; all statistical tests were 2-tailed, with a *P* value less than .05 considered statistically significant.

## Results

### Study characteristics

**Figure 1.**
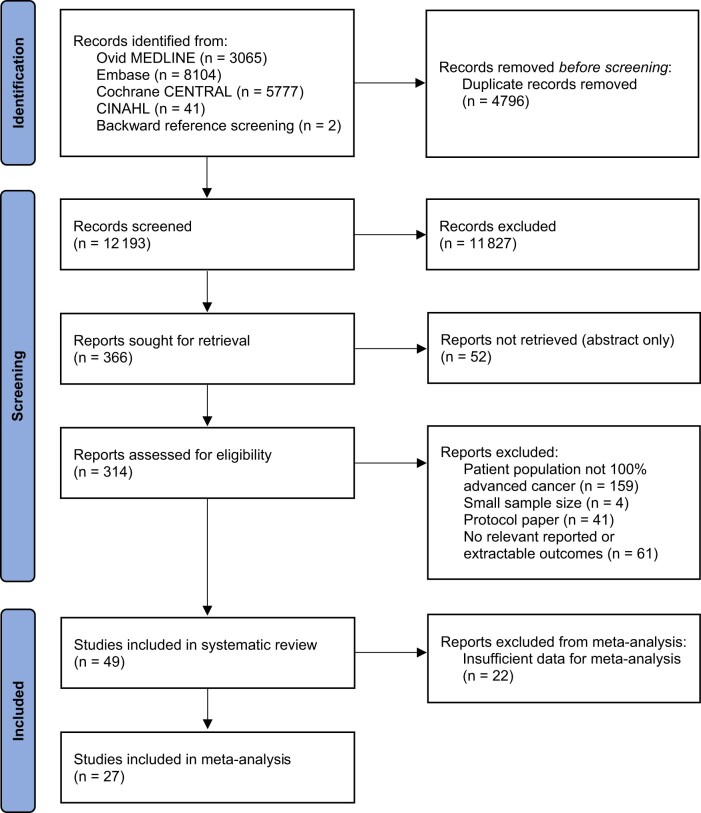
Preferred Reporting Items for Systematic reviews and Meta-Analyses flow diagram. CINAHL = Cumulative Index to Nursing and Allied Health Literature.

A total of 12 193 unique references were screened and 314 full-text articles were assessed; of these, 56 articles (41-96) reporting on outcomes of 49 trials for 8554 caregivers were ultimately included (Figure 1). Of the 49 included trials, 34 (69%) included caregivers of patients with mixed solid tumor malignancies, 6 (12%) only lung cancer (42,59-62,69,72,81), 1 (2%) breast cancer (74), 1 (2%) pancreatic cancer (94), 1 (2%) high-grade glioma (43), 2 (4%) gastrointestinal cancers (73,76), and 4 (8%) hematological malignancies (44,49,80,84). A total of 32 (65%) trials were conducted in an outpatient setting (42-45,53-57,59-77,81-83,85,87,88,91,92,94), 5 (10%) in a home setting (46-48,51,78,79,86), 4 (8%) in an inpatient setting (41,80,84,96), and 8 (16%) in mixed settings (49,50,52,58,89,90,93,95). Of the trials, 28 were conducted in the United States (44,45,49-51,53,55,59-68,71-77,83-86,89,92,94,95); 4 each in Australia (46-48,52,87,88) and China (69,80,81,96); 3 in Canada (42,70,91); 2 each in the United Kingdom (54,82), Netherlands (43,90), and Denmark (58,78,79); and 1 each in Colombia (41), Jordan (56), Korea (57), and Norway (93). Seven trials were at low risk of bias (42,44,60-62,65,66,84,85,91), 8 had some concerns for bias (47,48,55,59,63,64,74,76,77,83), and 34 were at high risk of bias (41,43,45,46,49-54,56-58,67-73,75,78-82,86-90,92-96) (Supplementary Figure 1, available online). There was concern for publication bias for the outcome of overall QOL (*P* =* *.009) (Supplementary Figure 2, available online).

A total of 16 (33%) trials reported on interventions directed at caregivers (41-57), 19 (39%) on interventions directed at the patient–caregiver dyad (58-81), and 14 (29%) on interventions directed at patients and/or their families (83-96). Most trials directed at caregivers evaluated psychoeducational or problem-solving interventions (13 of 16, 81%) (41-43,45-50,52,53,55-57); most directed at dyads evaluated counseling or therapy interventions (18 of 19, 95%) (58,59,63-67,69-81); and most directed at patients evaluated palliative care team interventions (10 of 14, 71%) (83-89,91,93,94,96). A total of 42 (86%) trials compared 1 intervention to usual care (42-46,49,50,52-56,58,59,63-72,74,75,77-96), 2 (4%) compared 2 interventions to usual care (47,48,51), and 5 (10%) compared 2 or more interventions without a usual care arm (41,57,60-62,73,76). For most trials reporting on proportion of spousal caregivers, more than 50% of participants were patients’ spouses (31 of 36, 86%) (42,44-49,52-55,58,59,63-66,68-73,75-77,83-85,90-94); for most reporting on caregiver sex, more than 60% were female caregivers (30 of 42, 71%) (41-51,54,55,57-59,65-69,73,76,78,79,83-86,90-93); and for most conducted in the United States and western Europe reporting on race, more than 70% of caregivers were White or Caucasian (15 of 18, 83%) (44,45,54,55,65-67,71-73,75,76,84,85,92,94). Other aspects of the study design and outcomes are reported in Supplementary Tables 1-3 (available online).

### Quality of life

**Figure 2.**
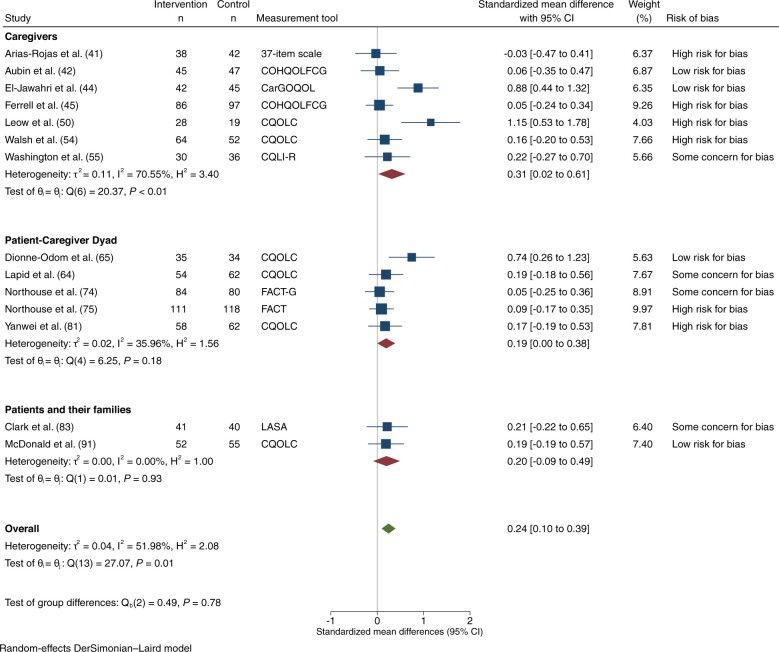
Overall quality of life, at 1-3 months. CI = confidence interval; CarGOQOL = Caregiver Oncology Quality of Life Questionnaire; COHQOLFCG = City of Hope Quality of Life Family Caregiver Version; CQLI-R = Caregiver Quality of Life Index-Revised; CQOLC = Caregiver Quality of Life Index-Cancer; FACT = Functional Assessment of Cancer Therapy; FACT-G = Functional Assessment of Cancer Therapy-General; LASA = linear analog self-assessment items.

QOL and/or its individual domains were assessed in 23 trials; 16 assessed overall QOL, 10 physical well-being, 8 mental well-being, and 6 other QOL subscales. A total of 16 trials assessed overall QOL: 5 at low risk of bias (42,44,65,66,84,91), 3 with some concerns (55,63,64,83), and 8 at high risk (41,45,50,51,54,56,57,81). Ten were directed at caregivers (41,42,44,45,50,51,54-57), 3 at the patient–caregiver dyad (64-66,81), and 3 at patients and/or their families (83,84,91). Fourteen trials involving 2264 participants had data that could be pooled for meta-analysis of overall QOL: 14 at 1-3 months (41,42,44,45,50,54,55,63-66,74,75,81,83,91) and 4 at 4- to 6-month follow-up (42,74,75,91). Of those measuring QOL at 1-3 months, 4 were at low risk (42,44,65,66,91), 4 had some concerns (55,63,64,74,83), and 6 at high risk of bias (41,45,50,54,55,75,81). There was a statistically significant effect on QOL at 1-3 months (SMD = 0.24, 95% [confidence interval] CI = 0.10 to 0.39; *I*^2^ = 52.0%; Figure 2), albeit with high risk of publication bias (Supplementary Figure 2, available online). There were no differences among subgroups by intervention target or by risk of bias. Among the 4 trials evaluating QOL at 4-6 months, there was no statistically significant improvement in QOL (Supplementary Figure 3.1, available online). Sensitivity analyses substituting the extensive for the brief intervention in 1 study (75) yielded similar conclusions.

### Physical well-being

**Figure 3.**
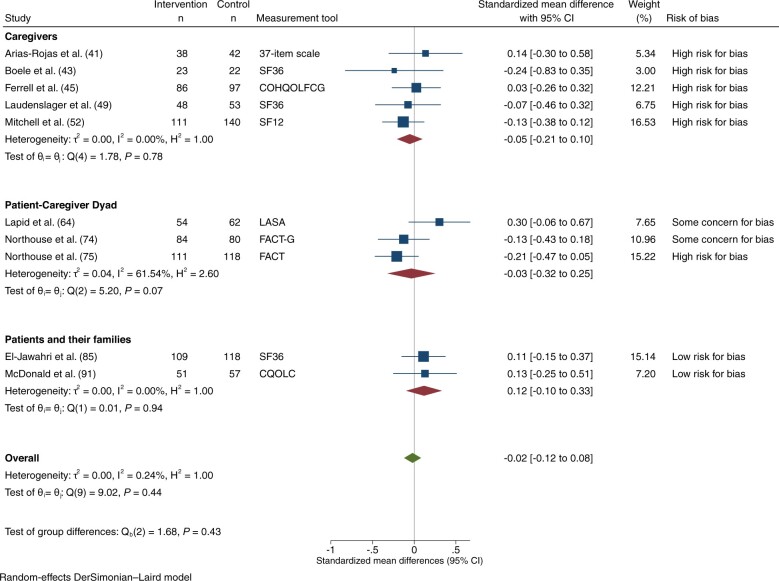
Physical well-being, at 1-3 months. COHQOLFCG = City of Hope Quality of Life Family Caregiver Version; CQOLC = Caregiver Quality of Life Index-Cancer; FACT = Functional Assessment of Cancer Therapy; FACT-G = Functional Assessment of Cancer Therapy-General; LASA = linear analog self-assessment items; SF12 = 12-Item Short-Form Health Survey; SF36 = 36-Item Short-Form Health Survey.

Physical well-being was reported in 11 trials: 2 at low risk of bias (85,91), 2 with some concerns (63,64,74), and 7 at high risk (41,45,49,52,58,71,75). Four were directed at caregivers (41,45,49,52), 5 at the patient–caregiver dyad (58,63,64,71,74,75), and 2 at patients and/or their families (85,91). A total of 11 trials involving 2226 participants had data on physical well-being that could be extracted and pooled for meta-analysis: 10 trials at 1-3 months (41,43,45,49,52,63,64,74,75,85,91) and 7 at 4-6 months (43,52,58,74,75,85,91). For those assessing physical well-being at 1-3 months, risk of bias was rated low for 2 trials (85,91), some concerns for 2 (63,64,74), and high for 6 (41,43,45,49,52,75). There was no statistically significant effect on physical well-being at 1-3 months (SMD = -0.02, 95% CI = -0.12 to 0.08; *I*^2^ = 0.2%; Figure 3), with no differences among subgroups by intervention target or by risk of bias. At 4-6 months, there was no effect on physical well-being (Supplementary Figure 3.2, available online). Results were similar in sensitivity analyses substituting the extensive for the brief intervention in 1 study (75).

### Mental well-being

**Figure 4.**
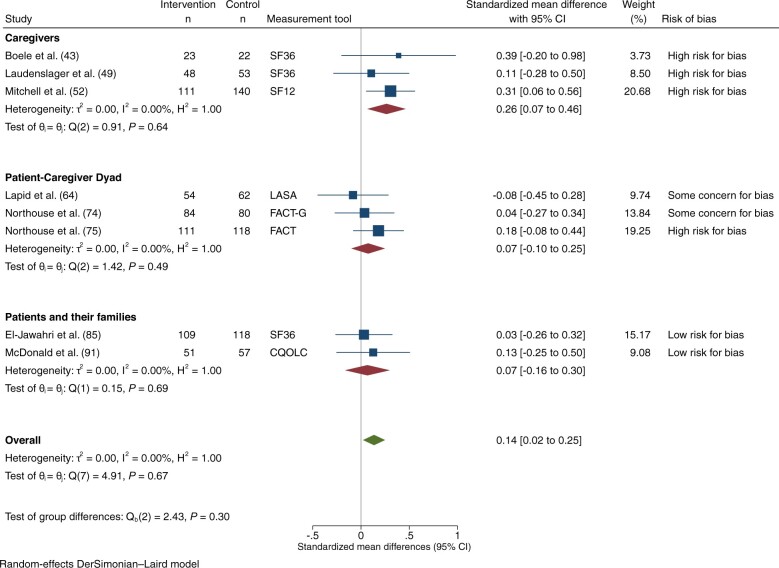
Mental well-being, at 1-3 months. CI = confidence interval; CQOLC = Caregiver Quality of Life Index-Cancer; FACT = Functional Assessment of Cancer Therapy; FACT-G = Functional Assessment of Cancer Therapy-General; LASA = linear analog self-assessment items; SF12 = 12-Item Short-Form Health Survey; SF36 = 36-Item Short-Form Health Survey.

Mental well-being was reported in 9 trials: 2 at low risk of bias (85,91), 2 with some concerns (63,64,74), and 5 at high risk (43,49,52,58,75). Nine trials, reporting on 1906 participants, had extractable data on mental well-being that could be pooled for meta-analysis (43,49,52,58,63,64,74,75,85,91): 8 at 1-3 months (43,49,52,63,64,74,75,85,91) and 7 at 4-6 months (43,52,58,74,75,85,91). Among those assessing mental well-being at 1-3 months, 2 were at low risk (85,91), 2 had some concerns (63,64,74), and 4 were at high risk of bias (43,49,52,75). There was a statistically significant effect on mental well-being at 1-3 months (SMD = 0.14, 95% CI = 0.02 to 0.25; *I*^2^ = 0.0%; Figure 4), with no statistically significant differences among subgroups by intervention target or by risk of bias. At 4-6 months, there was no statistically significant effect on mental well-being (Supplementary Figure 3.3, available online). Results were similar in a sensitivity analysis substituting the extensive for the brief intervention in 1 study (75).

### Anxiety

**Figure 5.**
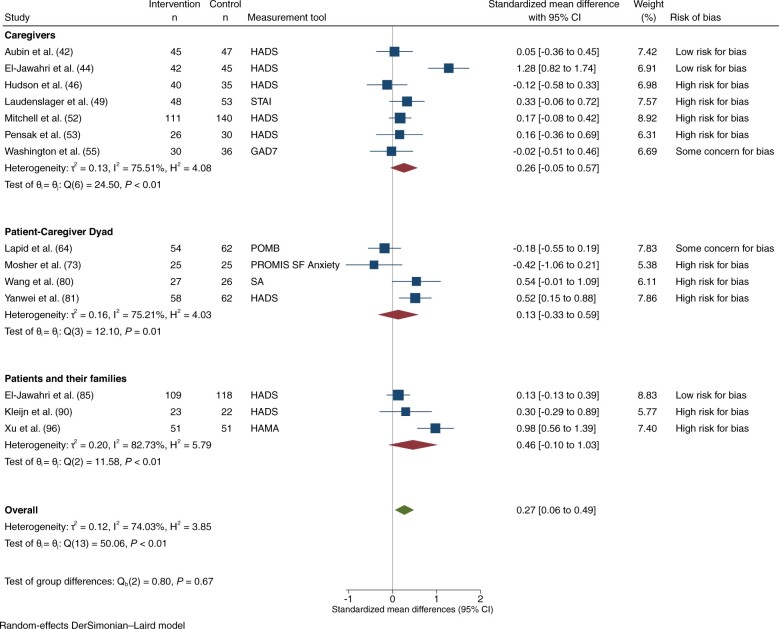
Anxiety, at 1-3 months. CI = confidence interval; HADS = Hospital Anxiety and Depression Scale; HAMA = Hamilton Anxiety Scale; GAD7 = General Anxiety Disorder-7; POMB = Profile of Mood States-B; PROMIS SF Anxiety = 6-Item Patient Reported Outcomes Measurement Information System Short-Form Anxiety Measure; SA = Social Anxiety Scale; STAI = State-Trait Anxiety Inventory.

Anxiety was assessed in 21 trials: 4 were at low risk of bias (42,44,84,85), 3 had some concerns (55,59,63,64), and 14 were at high risk (46,49,52,53,57,58,73,80-82,86,90,94,96). Eight trials had interventions directed at caregivers (42,44,46,49,52,53,55,57), 6 at the patient–caregiver dyad (58,59,63,64,73,80,81), and 7 at patients and/or their families (82,84-86,90,94,96). A total of 15 trials with 1874 participants had extractable data on anxiety for meta-analysis: 14 at 1-3 months (42,44,46,49,52,53,55,63,64,73,80,81,85,90,96) and 4 at 4-6 months (42,52,58,85). Among trials reporting on anxiety at 1-3 months, 3 were at low risk of bias (42,44,85), 2 had some concerns (55,63,64), and 9 were at high risk of bias (46,49,52,53,73,80,81,90,96). There was a statistically significant effect on anxiety at 1-3 months (SMD = 0.27, 95% CI = 0.06 to 0.49; *I*^2^ = 74.0%; Figure 5). There were no statistically significant differences among subgroups by intervention target or by risk of bias. Among the 4 studies reporting on anxiety at 4-6 months, there was no statistically significant effect on anxiety (Supplementary Figure 3.4, available online).

### Depression

**Figure 6.**
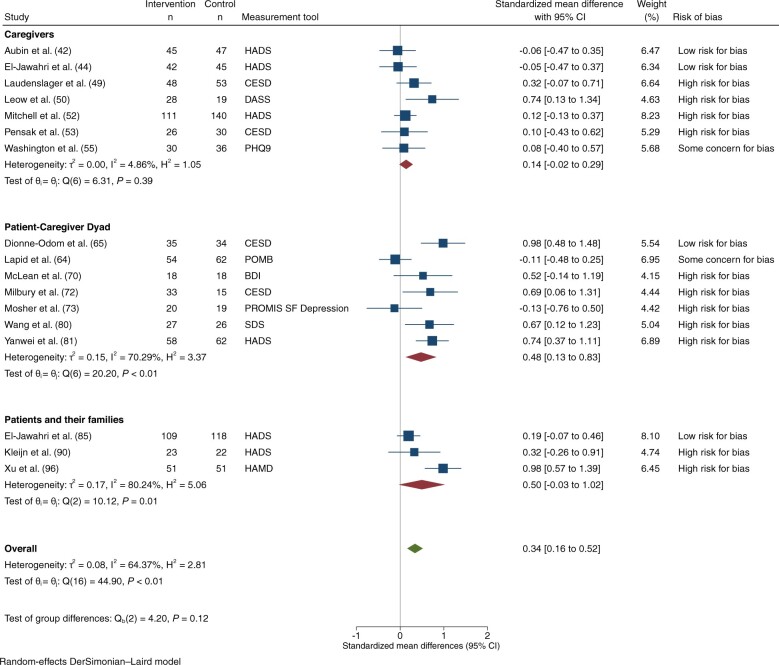
Depression, at 1-3 Months. BDI = Beck Depression Inventory; CESD = Center for Epidemiologic Studies-Depression; CI = confidence interval; DASS = Depression Anxiety Stress Scale; HADS = Hospital Anxiety and Depression Scale; HAMD = Hamilton Depression Scale; PHQ-9 = Patient Health Questionnaire-9; POMB = Profile of Mood States-B; PROMIS SF Depression = 6-Item Patient Reported Outcomes Measurement Information System Short-Form Depression Measure; SDS = Self-rating Depression Scale.

Depression was assessed in 27 trials: 4 at low risk of bias (42,44,65,66,84), 4 with some concerns (55,59,63,64,77), and 19 at high risk (49,50,52,53,57,58,70,72,73,80-82,86-90,94-96). Interventions were directed at caregivers in 8 trials (42,44,49,50,52,53,55,57), patient–caregiver dyads in 10 (58,59,63-66,70,72,73,77,80,81), and patients and/or their families in 9 (82,84,86-90,94-96). Eighteen studies involving 2087 participants had extractable data on depression for meta-analysis: 17 at 1-3 months (42,44,49,50,52,53,55,63-66,70,72,73,80,81,85,90,96) and 4 at 4-6 months (42,52,58,85). Among trials reporting at 1-3 months, 4 were at low risk of bias (42,44,65,66,85), 2 at some concerns (55,63,64), and 11 at high risk (49,50,52,53,70,72,73,80,81,90,96). There was a statistically significant effect on depression at 1-3 months (SMD = 0.34, 95% CI = 0.16 to 0.52; *I*^2^ = 64.4%; Figure 6). Although the difference between subgroups was not statistically significant (*P* =* *.12), interventions directed at patient–caregiver dyads tended to be associated with the greatest improvement in depression (SMD = 0.48, 95% CI = 0.13 to 0.83; *I*^2^ = 70.3). There was a similar trend for interventions directed at patients and/or their families, but there were only 3 trials in this subgroup and results were not statistically significant. Most trials were at high risk of bias, with this larger group also tending to have the greatest improvement in depression (*P* = .03). At 4-6 months, there was no statistically significant effect on depression (Supplementary Figure 3.5, available online).

### Other outcomes

Caregiver burden was reported in 20 trials: 3 at low risk of bias (44,60-62,65,66), 3 with some concerns (59,77,83), and 14 at high risk (45,49-51,53,54,67,70,73,90,92,94-96). Seven reported on interventions directed at caregivers (44,45,49-51,53,54), 7 at the patient–caregiver dyad (59-62,65-67,70,73,77), and 6 at patients and/or their families (83,90,92,94-96). Statistically significant results favoring the intervention were reported for 8 trials of which 2 were at low risk of bias (44,65,66), 2 were directed at caregivers (44,49), 5 at dyads (59,65,66,70,73), and 1 at patients and/or their families (96). No differences were noted between trial groups for the other 24 endpoints related to caregiver burden.

Caregiver self-efficacy was reported in 15 trials (42-48,50,51,59-62,67,73,75-77): 3 at low risk of bias (42,44,60-62), 4 with some concerns (47,48,59,76,77), and 8 at high risk (43,45,46,50,51,67,73,75). Eight trials studied interventions directed at caregivers (42-48,50,51), and 7 studied interventions directed at the patient–caregiver dyad (59-62,67,73,75-77). Statistically significant results favoring the intervention were reported for 9 trials of which 2 were at low risk of bias (42,44); 5 were directed at caregivers (42-45,50), and 4 at dyads (59,67,75,77).

Family functioning was reported in 7 trials (47,48,69,70,76,80,81,87,88): 4 with some concerns for bias (47,48,69,70,76) and 3 at high risk (80,81,87,88). One trial reported on an intervention directed at caregivers (47,48), 5 at patient–caregiver dyads (69,70,76,80,81), and 1 at patients and/or their families (87,88). Three trials reported improved family functioning related to the intervention; all 3 were at high risk of bias and directed at dyads (69,70,80).

Bereavement outcomes of depression or grief were reported in 5 trials (54,65,66,87-89,93): 1 at low risk (65,66) and 4 at high risk of bias (54,87-89,93). One reported on an intervention directed at caregivers (54), 1 at the patient–caregiver dyad (65,66), and 3 at patients and/or their families (87-89,93). Two studies reported improved grief (89,93), and none improved depression postbereavement.

## Discussion

In this systematic review and meta-analysis of 49 randomized controlled trials, interventions for caregivers resulted in improvements at 1-3 months in caregiver QOL, mental well-being, anxiety, and depression but not in physical well-being. The longer-term impact of these interventions is uncertain because of the lack of statistically significant findings at 4-6 months in the few studies with outcome data at this endpoint. In the narrative synthesis, interventions led to improvements in caregiver self-efficacy, with mixed findings for caregiver burden, family functioning, and bereavement outcomes.

To our knowledge, this is the first meta-analysis that reports specifically on studies of interventions for caregivers of patients with advanced cancer; our results provide substantive evidence for the benefit of interventions for this vulnerable population. The last large meta-analysis of interventions for family caregivers of patients with cancer was in 2010 and focused mostly on those caring for patients at earlier stages of cancer (19). Statistically significant effects were observed for caregiver burden, coping, and self-efficacy, as well as for a combined outcome of anxiety, distress, and mood but not for depression or physical QOL; risk of bias assessment was not performed. Of note, depression was low at baseline, which might have been due to the inclusion mainly of patients at earlier disease stages. Subsequent reviews summarized results for recent trials but were unable to make definitive conclusions because of lack of meta-analysis and risk of bias assessment (15,97) or because of restrictive inclusion criteria yielding small samples (21-23). Our review adds to these results by providing a comprehensive meta-analysis including 29 trials (41-44,49,52,53,57,67-69,72,73,76,77,80-90,92,93,95,96) that were not included in recent publications (21-23). In addition, our meta-analysis focuses specifically on trials of interventions for caregivers of patients with advanced cancer, who are in particular need of support due to having worse QOL, mental health, anxiety, and depression than those caring for patients at earlier stages of the disease trajectory (6,15). Moreover, we have evaluated the risk of bias of each trial and included subgroup analyses according to the target of the intervention.

Our meta-analysis demonstrated a modest effect from caregiver interventions on overall QOL and mental well-being but not on physical well-being. Although many trials used QOL measures developed and validated for caregivers, these measures lack items relevant to the advanced cancer setting, which might have attenuated the effect in this population (98). Mental well-being was measured mostly using subscales of QOL measures designed for general populations or for patients and included items on social functioning and vitality in addition to mental health items (99,100). Development and validation of caregiver QOL measures specifically for the advanced cancer setting would be a valuable contribution to this area of research. The lack of impact on physical well-being may be related to the relatively good physical well-being of many caregivers compared with general population samples (6,10). As well, our review explicitly focused on psychoeducational, skills training, counseling, or team-based interventions focusing on caregiving or coping; most of these interventions had a psychoeducational and/or psychotherapeutic focus. Interventions with a focus on individual symptoms such as sleep might be more likely to improve physical well-being, but these were excluded because of being outside the focus of the current review.

The effects of caregiver interventions on outcomes of depression and anxiety are noteworthy because these are important mental health conditions for which the prevalence in advanced care settings is as great or greater among caregivers than among the patients they care for (9,15,101). High levels of depression and anxiety in this population may reflect the considerable symptom burden and care needs of patients with advanced disease, lack of preparation of caregivers for their role, and grief due to current and anticipated relational losses (8,102-104). Of note, improvements in self-efficacy were reported for most interventions in this review, which might have played a part in alleviating anxiety and depression (11).

Results of the subgroup analyses according to intervention target demonstrated no statistically significant subgroup effects. The most substantial subgroup differences were observed for depression for which interventions directed at patient–caregiver dyads (mainly using counseling or therapy interventions) had the greatest effect. Relational factors that have been associated with depression in caregivers include a spousal patient–caregiver relationship and family or spousal conflict (8,11,105). Of the 6 trials targeting dyads with data meta-analyzed for depression that provided patient–caregiver relationship status (63-66,70,72,73,80), 5 (>70%) included mainly spouses or partners (63-66,70,72,73) and 1 included only couples with marital difficulties (70). As well, physical and emotional symptom distress are common in patients with advanced cancer and are associated with increased depression in their caregivers (11,15). Addressing concerns of the patient and caregiver simultaneously may contribute to alleviation of depression in caregivers receiving dyad interventions. To further assess the impact of intervention target and factors that may contribute to this impact, trials are needed that directly compare interventions targeting patients, caregivers, or both and that conduct analyses to explore factors that mediate or moderate the effects of different interventions.

This review has limitations. Most trials had less than 100 participants and were at high risk of bias, and standardized mean differences were small. Although we were strict in applying risk of bias criteria and ratings should be considered conservative, there are areas that could be improved for future trials. For many trials, bias was due to missing outcome data, as observed in previous reviews that assessed patient outcomes in advanced cancer (24,25). Availability of data from 95% of participants, as recommended by the Cochrane Collaboration, is rare for patients with advanced cancer and their caregivers because of high levels of distress and burden (34). Nevertheless, few studies used analysis methods that corrected for bias or conducted sensitivity analyses. Similar to previous reviews in advanced cancer or palliative care settings, bias in measurement of outcome data was noted for all studies, because outcomes were participant reported and it is not possible to blind participants receiving behavioral interventions (24,35). Although almost all trials included in our meta-analysis used validated measures, none were validated specifically for caregivers of adults with advanced cancer, and degree of adherence to the intervention (or lack thereof) was often not reported. In addition, there was diversity among studies in intervention design, measurement and reporting of outcomes, and countries and their health-care systems, which may have contributed to the high heterogeneity in several analyses. Most trials either did not report on participants’ race or ethnicity or included predominantly White or Caucasian caregivers. Additional trials with diverse samples are needed to provide conclusions with wider generalizability.

In this systematic review and meta-analysis, caregiver interventions resulted in improvements in QOL and mental health outcomes for caregivers of patients with advanced cancer. Further trials with large samples and longer follow-up are needed to substantiate these data and to delineate which interventions are most effective. To reduce risk of bias, investigators planning future studies should prespecify their analysis plan and register their trial prior to commencing recruitment; adhere to guidelines such as the Template for Intervention Description and Replication checklist to describe their intervention and adherence by participants (106); use measures validated for caregivers when these are available; and make a plan for handling missing data, including sensitivity analyses to demonstrate that data are little changed under a range of assumptions. Particular attention should be paid to diversity of participants. Although no specific intervention can be recommended over another at this time, cancer centers should ensure that interventions for caregivers are available; these could be in the form of psychoeducational or problem-solving interventions, couple-based counseling, or referral to an interdisciplinary palliative care team. Oncologists providing care for patients with advanced cancer should routinely ask about caregiver well-being and offer referral to available services.

## Supplementary Material

djad075_Supplementary_Data

## Data Availability

The data underlying this article are available in the article and in its online supplementary material.
