# Supplementary material for: Interventions to improve outcomes for caregivers of patients with advanced cancer: a meta-analysis
Source: J Natl Cancer Inst. 2023 Jun 5;115(8):896–908. doi: 10.1093/jnci/djad075 (PMC10407714; doi:10.1093/jnci/djad075)
Supplement: djad075_Supplementary_Data [file djad075_supplementary_data.pdf]

Supplementary Material

Supplementary Methods 1. Search Strategy

Supplementary Methods 2. Study Inclusion and Exclusion Criteria

Supplementary Methods 3. Methodological Details Regarding Risk of Bias Assessment

Supplementary Table 1. Study Characteristics, of Interventions Directed at Caregivers

Supplementary Table 2. Study Characteristics, of Interventions Directed at Patient-Caregiver Dyad

Supplementary Table 3. Study Characteristics, of Interventions Directed at Patients and Their Families

Supplementary Figure 1. Cochrane Risk of Bias Assessment. 1.1 By Study; 1.2 Summary

Supplementary Figure 2. Funnel Plots, for Assessment of Publication Bias at 1-3 Months. 2.1 Overall Quality of Life (p=0.039); 2.2 Physical Well-being (p=0.018); 2.3 Mental Well-being (p=0.042); 2.4 Anxiety (p=0.828); 2.5 Depression (p=0.051)

Supplementary Figure 3. Caregiver-Reported Outcomes at 4-6 Months. 3.1 Overall Quality of Life; 3.2 Physical Well-Being; 3.3 Mental Well-Being; 3.4 Anxiety; 3.5 Depression

Supplementary Methods 1. Search Strategy

Database: Ovid MEDLINE(R) and Epub Ahead of Print, In-Process & Other Non-Indexed Citations, Daily
and Versions(R) <1946 to June 14, 2021> Search Strategy:

----

1 exp Neoplasms/ (3480493)
2 (cancer or neoplasm* or carcinoma or tumor or tumour).mp. (4142314)
3 or/1-2 (4566582)
4 exp Caregivers/ (40010)
5 exp Family/ (332099)
6 exp Spouses/ (10620)
7 (caregiver* or "care giver*" or carer* or spouse* or husband* or wife or wives or sibling* or brother* or sister* or partner* or parent* or family or clerg* or supportive care or ((people or individual*) adj "caring for patient*")).mp. (1806474)
8 or/4-7 (1874499)
9 exp "Quality of Life"/ (212711)
10 ("quality of life" or "quality of lives").mp. (376337)
11 exp Mental Health/ (44112)
12 exp Mental Health Services/ (99007)
13 exp Mental Disorders/ (1292281)
14 mental health.mp. (213934)
15 exp Depression/ (128501)
16 exp Depressive Disorder/ (112964)
17 (depression or depressive or affective).mp. (537844)
18 exp Anxiety/ (92468)
19 exp Anxiety Disorders/ (82143)
20 anxiety.mp. (257566)
21 distress.mp. (144080)
22 exp Adaptation, Psychological/ (131291)
23 (cope* or coping).mp. (109547)
24 burden.mp. (253218)
25 exp Bereavement/ (13790)
26 bereavement.mp. (9111)
27 exp Grief/ (9116)
28 (grief* or griev*).mp. (15421)
29 stress.mp. (989082)
30 exp Stress, Psychological/ (138837)
31 exp Adaptation, Psychological/ (131291)
32 exp Self Efficacy/ (21646)
33 self efficacy.mp. (39710)
34 exp Self Concept/ (114116)
35 (confidence or confident).mp. (574612)
36 mastery.mp. (6670)
37 (preparedness or prepared).mp. (440593)
38 appraisal.mp. (40827)

39 or/9-38 (4316905)
40 3 and 8 and 39 (44845)
41 limit 40 to clinical trial, all (2274)
42 ((randomized or randomised or controlled) adj2 (trial or study or experiment)).ti. (179573)
43 Randomized controlled trial/ or Randomized controlled trials as Topic/ or Random allocation/ or Double blind method/ or Single blind method/ (791179)
44 Clinical trial/ or Clinical Trials as Topic/ (655124)
45 ((singl$ or doubl$ or treb$ or tripl$) adj (blind$3 or mask$3)).tw. or Placebos/ or Placebo$.tw. or Randomly allocated.tw. or (allocat$ adj3 random$).tw. or random*.tw. or blind*.tw. (1439182)
46 Case reports/ or Letter/ or Historical article/ (3461565)
47 (42 or 43 or (44 and 45)) not 46 (854200)
48 40 and 47 (2552)
49 41 or 48 (3165)
50 limit 49 to english language (3065)

Database: Embase Classic+Embase <1947 to 2021 Week 23> Search Strategy:
----
1 exp neoplasm/ (5192218)
2 (cancer or neoplasm* or carcinoma or tumor or tumour).mp. (5819858)
3 or/1-2 (6516360)
4 exp caregiver/ (90122)
5 exp caregiver burden/ (8557)
6 exp Caregiver Strain Index/ (296)
7 exp caregiver support/ (3661)
8 exp family/ (589087)
9 exp spouse/ (21384)
10 (caregiver* or "care giver*" or carer* or spouse* or husband* or wife or wives or sibling* or brother* or sister* or partner* or parent* or family or clerg* or supportive care or ((people or individual*) adj "caring for patient*")).mp. (2285571)
11 or/4-10 (2444749)
12 exp "quality of life"/ (534974)
13 ("quality of life" or "quality of lives").mp. (651517)
14 exp mental health/ (181495)
15 exp mental health service/ (62620)
16 exp mental disease/ (2463699)
17 mental health.mp. (328639)
18 exp depression/ (518944)
19 (depression or depressive or affective).mp. (831894)
20 exp anxiety/ (240869)
21 exp anxiety disorder/ (267741)
22 exp anxiety assessment/ (50196)
23 anxiety.mp. (412911)
24 exp distress syndrome/ (49529)

25 distress.mp. (248244)
26 exp coping behavior/ (70884)
27 (cope* or coping).mp. (164406)
28 exp caregiver burden/ (8557)
29 burden.mp. (369884)
30 exp caregiver strain index/ (296)
31 exp bereavement/ (9598)
32 exp bereavement support/ (886)
33 bereavement.mp. (12373)
34 exp grief/ (14131)
35 (grief* or griev*).mp. (17505)
36 exp stress/ (337445)
37 exp mental stress/ (90876)
38 stress.mp. (1396947)
39 exp self concept/ (220223)
40 self efficacy.mp. (38235)
41 (confidence or confident).mp. (720386)
42 mastery.mp. (8403)
43 (preparedness or prepared).mp. (566324)
44 appraisal.mp. (47696)
45 or/12-44 (6280356)
46 3 and 11 and 45 (88860)
47 limit 46 to (clinical trial or randomized controlled trial or controlled clinical trial or multicenter study or phase 1 clinical trial or phase 2 clinical trial or phase 3 clinical trial or phase 4 clinical trial) (8102)
48 Randomized controlled trial/ (663183)
49 ((randomized or randomised or controlled) adj2 (trial or study)).ti. (229062)
50 Clinical trial/ (1025746)
51 Randomization/ or Single blind procedure/ or Double blind procedure/ or Crossover procedure/ or Placebo/ or Randomi?ed controlled trial$.tw. or Rct.tw. or Random allocation.tw. or Randomly allocated.tw. or Allocated randomly.tw. or (allocated adj2 random).tw. or Single blind$.tw. or Double blind$.tw. or ((treble or triple) adj blind$).tw. or Placebo$.tw. or Prospective study/ (1611480)
52 50 and 51 (348108)
53 Case study/ or Case report/ or Abstract report/ or letter/ (3784340)
54 (48 or 49 or 52) not 53 (860475)
55 46 and 54 (3994)
56 47 or 55 (8277)
57 limit 56 to english language (8104)

Database: EBM Reviews - Cochrane Central Register of Controlled Trials <May 2021>
Search Strategy:
1 exp Neoplasms/ (82227)
2 (cancer or neoplasm* or carcinoma or tumor or tumour).mp. (233102)
3 or/1-2 (243261)

4 exp Caregivers/ (2230)
5 exp Family/ (9686)
6 exp Spouses/ (374)
7 (caregiver* or "care giver*" or carer* or spouse* or husband* or wife or wives or sibling* or brother* or sister* or partner* or parent* or family or clerg* or supportive care or ((people or individual*) adj "caring for patient*")).mp. (105682)
8 or/4-7 (107224)
9 exp "Quality of Life"/ (25037)
10 ("quality of life" or "quality of lives").mp. (121067)
11 exp Mental Health/ (1634)
12 exp Mental Health Services/ (6958)
13 exp Mental Disorders/ (72096)
14 mental health.mp. (22974)
15 exp Depression/ (12808)
16 exp Depressive Disorder/ (12610)
17 (depression or depressive or affective).mp. (93277)
18 exp Anxiety/ (8209)
19 exp Anxiety Disorders/ (9438)
20 anxiety.mp. (56669)
21 distress.mp. (22939)
22 exp Adaptation, Psychological/ (5421)
23 (cope* or coping).mp. (13667)
24 burden.mp. (24596)
25 exp Bereavement/ (231)
26 bereavement.mp. (483)
27 exp Grief/ (132)
28 (grief* or griev*).mp. (645)
29 stress.mp. (64656)
30 exp Stress, Psychological/ (6222)
31 exp Adaptation, Psychological/ (5421)
32 exp Self Efficacy/ (3253)
33 self efficacy.mp. (14276)
34 exp Self Concept/ (6869)
35 (confidence or confident).mp. (87309)
36 mastery.mp. (1047)
37 (preparedness or prepared).mp. (12232)
38 appraisal.mp. (2149)
39 or/9-38 (434345)
40 3 and 8 and 39 (5777)

Database: CINAHL

Search ID#	Search Terms	Search Options	Last Run Via	Results
S59	S3 AND S18 AND S58	Limiters - English Language; Publication Type: Clinical Trial, Randomized Controlled Trial<br>Search modes - Boolean/Phrase	Interface - EBSCOhost Research Databases<br>Search Screen - Advanced Search<br>Database - CINAHL	1,295
S58	S19 or S20 or S21 or S22 or S23 or S24 or S25 or S26 or S27 or S28 or S29 or S30 or S31 or S32 or S33 or S34 or S35 or S36 or S37 or S38 or S39 or S40 or S41 or S42 or S43 or S44 or S45 or S46 or S47 or S48 or S49 or S50 or S51 or S52 or S53 or S54 or S55 or S56 or S57	Search modes - Boolean/Phrase	Interface - EBSCOhost Research Databases<br>Search Screen - Advanced Search<br>Database - CINAHL	1,355,505
S57	TX appraisal	Search modes - Boolean/Phrase	Interface - EBSCOhost Research Databases<br>Search Screen - Advanced Search<br>Database - CINAHL	21,298
S56	TX (preparedness or prepared)	Search modes - Boolean/Phrase	Interface - EBSCOhost Research Databases<br>Search Screen - Advanced Search<br>Database - CINAHL	38,952
S55	TX mastery	Search modes - Boolean/Phrase	Interface - EBSCOhost Research Databases<br>Search Screen - Advanced	3,746

			Search<br>Database - CINAHL	
S54	(MH "Empowerment")	Search modes - Boolean/Phrase	Interface - EBSCOhost Research Databases<br>Search Screen - Advanced Search<br>Database - CINAHL	11,430
S53	TX (confidence or confident)	Search modes - Boolean/Phrase	Interface - EBSCOhost Research Databases<br>Search Screen - Advanced Search<br>Database - CINAHL	311,999
S52	(MH "Confidence")	Search modes - Boolean/Phrase	Interface - EBSCOhost Research Databases<br>Search Screen - Advanced Search<br>Database - CINAHL	7,847
S51	TX self concept	Search modes - Boolean/Phrase	Interface - EBSCOhost Research Databases<br>Search Screen - Advanced Search<br>Database - CINAHL	28,586
S50	(MH "Self Concept+")	Search modes - Boolean/Phrase	Interface - EBSCOhost Research Databases<br>Search Screen - Advanced Search<br>Database - CINAHL	56,444
S49	TX self efficacy	Search modes - Boolean/Phrase	Interface - EBSCOhost Research Databases<br>Search Screen - Advanced	26,104

			Search<br>Database - CINAHL	
S48	(MH "Self-Efficacy")	Search modes - Boolean/Phrase	Interface - EBSCOhost Research Databases<br>Search Screen - Advanced Search<br>Database - CINAHL	18,216
S47	TX stress	Search modes - Boolean/Phrase	Interface - EBSCOhost Research Databases<br>Search Screen - Advanced Search<br>Database - CINAHL	185,153
S46	(MH "Adaptation, Psychological+")	Search modes - Boolean/Phrase	Interface - EBSCOhost Research Databases<br>Search Screen - Advanced Search<br>Database - CINAHL	29,626
S45	(MH "Stress, Psychological+")	Search modes - Boolean/Phrase	Interface - EBSCOhost Research Databases<br>Search Screen - Advanced Search<br>Database - CINAHL	71,349
S44	(MH "Stress+")	Search modes - Boolean/Phrase	Interface - EBSCOhost Research Databases<br>Search Screen - Advanced Search<br>Database - CINAHL	82,729
S43	TX (grief* or griev*)	Search modes - Boolean/Phrase	Interface - EBSCOhost Research Databases<br>Search Screen - Advanced	12,511

			Search<br>Database - CINAHL	
S42	(MH "Anticipatory Grieving")	Search modes - Boolean/Phrase	Interface - EBSCOhost Research Databases<br>Search Screen - Advanced Search<br>Database - CINAHL	138
S41	(MH "Grief+")	Search modes - Boolean/Phrase	Interface - EBSCOhost Research Databases<br>Search Screen - Advanced Search<br>Database - CINAHL	7,152
S40	TX bereavement	Search modes - Boolean/Phrase	Interface - EBSCOhost Research Databases<br>Search Screen - Advanced Search<br>Database - CINAHL	8,395
S39	(MH "Bereavement+")	Search modes - Boolean/Phrase	Interface - EBSCOhost Research Databases<br>Search Screen - Advanced Search<br>Database - CINAHL	12,290
S38	TX burden	Search modes - Boolean/Phrase	Interface - EBSCOhost Research Databases<br>Search Screen - Advanced Search<br>Database - CINAHL	67,401
S37	(MH "Caregiver Burden")	Search modes - Boolean/Phrase	Interface - EBSCOhost Research Databases<br>Search Screen - Advanced	8,594

			Search<br>Database - CINAHL	
S36	TX (cope* or coping)	Search modes - Boolean/Phrase	Interface - EBSCOhost Research Databases<br>Search Screen - Advanced Search<br>Database - CINAHL	84,689
S35	(MH "Family Coping")	Search modes - Boolean/Phrase	Interface - EBSCOhost Research Databases<br>Search Screen - Advanced Search<br>Database - CINAHL	1,926
S34	(MH "Coping+")	Search modes - Boolean/Phrase	Interface - EBSCOhost Research Databases<br>Search Screen - Advanced Search<br>Database - CINAHL	31,337
S33	TX distress	Search modes - Boolean/Phrase	Interface - EBSCOhost Research Databases<br>Search Screen - Advanced Search<br>Database - CINAHL	48,317
S32	(MH "Family Distress Index")	Search modes - Boolean/Phrase	Interface - EBSCOhost Research Databases<br>Search Screen - Advanced Search<br>Database - CINAHL	2
S31	TX anxiety	Search modes - Boolean/Phrase	Interface - EBSCOhost Research Databases<br>Search Screen - Advanced	89,671

			Search<br>Database - CINAHL	
S30	(MH "Anticipatory Anxiety")	Search modes - Boolean/Phrase	Interface - EBSCOhost Research Databases<br>Search Screen - Advanced Search<br>Database - CINAHL	434
S29	(MH "Anxiety Disorders+")	Search modes - Boolean/Phrase	Interface - EBSCOhost Research Databases<br>Search Screen - Advanced Search<br>Database - CINAHL	39,029
S28	(MH "Anxiety+")	Search modes - Boolean/Phrase	Interface - EBSCOhost Research Databases<br>Search Screen - Advanced Search<br>Database - CINAHL	37,895
S27	TX (depression or depressive or affective)	Search modes - Boolean/Phrase	Interface - EBSCOhost Research Databases<br>Search Screen - Advanced Search<br>Database - CINAHL	177,882
S26	(MH "Depression+")	Search modes - Boolean/Phrase	Interface - EBSCOhost Research Databases<br>Search Screen - Advanced Search<br>Database - CINAHL	96,634
S25	TX mental health	Search modes - Boolean/Phrase	Interface - EBSCOhost Research Databases<br>Search Screen - Advanced	188,811

			Search<br>Database - CINAHL	
S24	(MH "Mental Disorders+")	Search modes - Boolean/Phrase	Interface - EBSCOhost Research Databases<br>Search Screen - Advanced Search<br>Database - CINAHL	488,177
S23	(MH "Mental Health Services+")	Search modes - Boolean/Phrase	Interface - EBSCOhost Research Databases<br>Search Screen - Advanced Search<br>Database - CINAHL	68,823
S22	(MH "Mental Health")	Search modes - Boolean/Phrase	Interface - EBSCOhost Research Databases<br>Search Screen - Advanced Search<br>Database - CINAHL	29,376
S21	TX (quality of life or quality of lives)	Search modes - Boolean/Phrase	Interface - EBSCOhost Research Databases<br>Search Screen - Advanced Search<br>Database - CINAHL	152,851
S20	(MH "Quality of Life (Iowa NOC)")	Search modes - Boolean/Phrase	Interface - EBSCOhost Research Databases<br>Search Screen - Advanced Search<br>Database - CINAHL	3
S19	(MH "Quality of Life+")	Search modes - Boolean/Phrase	Interface - EBSCOhost Research Databases<br>Search Screen - Advanced	100,947

			Search<br>Database - CINAHL	
S18	S4 or S5 or S6 or S7 or S8 or S9 or S10 or S11 or S12 or S13 or S14 or S15 or S16 or S17	Search modes - Boolean/Phrase	Interface - EBSCOhost Research Databases<br>Search Screen - Advanced Search<br>Database - CINAHL	Display
S17	TX (caregiver* or "care giver*" or carer* or spouse* or husband* or wife or wives or sibling* or brother* or sister* or partner* or parent* or family or clerg* or supportive care or ((people or individual*) N1 "caring for patient*"))	Search modes - Boolean/Phrase	Interface - EBSCOhost Research Databases<br>Search Screen - Advanced Search<br>Database - CINAHL	Display
S16	(MH "Spouses")	Search modes - Boolean/Phrase	Interface - EBSCOhost Research Databases<br>Search Screen - Advanced Search<br>Database - CINAHL	Display
S15	(MH "Family+")	Search modes - Boolean/Phrase	Interface - EBSCOhost Research Databases<br>Search Screen - Advanced Search<br>Database - CINAHL	Display
S14	(MH "Caregiver Physical Health (Iowa NOC)")	Search modes - Boolean/Phrase	Interface - EBSCOhost Research Databases<br>Search Screen - Advanced Search<br>Database - CINAHL	Display

S13	(MH "Caregiver Role Strain (NANDA)")	Search modes - Boolean/Phrase	Interface - EBSCOhost Research Databases<br>Search Screen - Advanced Search<br>Database - CINAHL	Display
S12	(MH "Caregiver Strain Index")	Search modes - Boolean/Phrase	Interface - EBSCOhost Research Databases<br>Search Screen - Advanced Search<br>Database - CINAHL	Display
S11	(MH "Caregiver Well-Being (Iowa NOC)")	Search modes - Boolean/Phrase	Interface - EBSCOhost Research Databases<br>Search Screen - Advanced Search<br>Database - CINAHL	Display
S10	(MH "Caregiver-Patient Relationship (Iowa NOC)")	Search modes - Boolean/Phrase	Interface - EBSCOhost Research Databases<br>Search Screen - Advanced Search<br>Database - CINAHL	Display
S9	(MH "Caregiver Stressors (Iowa NOC)")	Search modes - Boolean/Phrase	Interface - EBSCOhost Research Databases<br>Search Screen - Advanced Search<br>Database - CINAHL	Display
S8	(MH "Caregiver Support")	Search modes - Boolean/Phrase	Interface - EBSCOhost Research Databases<br>Search Screen - Advanced Search<br>Database - CINAHL	Display

S7	(MH "Caregivers")	Search modes - Boolean/Phrase	Interface - EBSCOhost Research Databases<br>Search Screen - Advanced Search<br>Database - CINAHL	Display
S6	(MH "Caregiver Attitudes")	Search modes - Boolean/Phrase	Interface - EBSCOhost Research Databases<br>Search Screen - Advanced Search<br>Database - CINAHL	Display
S5	(MH "Caregiver Burden")	Search modes - Boolean/Phrase	Interface - EBSCOhost Research Databases<br>Search Screen - Advanced Search<br>Database - CINAHL	Display
S4	(MH "Family Caregiver Status (Iowa NOC)+")	Search modes - Boolean/Phrase	Interface - EBSCOhost Research Databases<br>Search Screen - Advanced Search<br>Database - CINAHL	Display
S3	S1 OR S2	Search modes - Boolean/Phrase	Interface - EBSCOhost Research Databases<br>Search Screen - Advanced Search<br>Database - CINAHL	Display
S2	TX (cancer or neoplasm* or carcinoma or tumor or tumour)	Search modes - Boolean/Phrase	Interface - EBSCOhost Research Databases<br>Search Screen - Advanced Search<br>Database - CINAHL	Display

S1	(MH "Neoplasms+")	Search modes - Boolean/Phrase	Interface - EBSCOhost Research Databases<br>Search Screen - Advanced Search<br>Database - CINAHL	Display

Supplementary Methods 2. Study Inclusion and Exclusion Criteria

**Inclusion Criteria**	**Exclusion Criteria**
Sample:<br>- Adult caregivers (≥ 18 years old) of adult patients (≥ 18 years old)<br>- All patients have advanced cancer (Stage 3 or 4 solid tumour diagnosis, or diagnosis of a hematologic malignancy where the 5-year survival is less than 50%)<br><br>Intervention:<br>- Psycho-educational, skills training, counseling or team-based interventions to offer direct or indirect support with caregiving or coping<br><br>Outcomes:<br>- Quality of life, depression, anxiety, general mental health, general physical health, caregiving outcomes (e.g. burden, self-efficacy, confidence, and preparedness for the caregiving role), and bereavement outcomes (e.g. grief)<br><br>Study Design:<br>- Randomized controlled trials	Sample:<br>- Some patients with early-stage cancer, or non-cancer diagnosis<br><br>Intervention:<br>- Complementary therapies (e.g. massage)<br>- Interventions specifically targeted at one symptom (e.g. insomnia)<br><br>Study Design:<br>- Non-randomized trial designs<br>- Trials with total n<20 for caregivers

Supplementary Methods 3. Methodological Details Regarding Risk of Bias Assessment

All studies were assessed for their risk of bias using the Cochrane Collaboration's Risk of Bias tool version 2. This tool contains five domains: risk of bias arising from the randomization process; due to deviations from the intended interventions; due to missing outcome data; from measurement of the outcome; or in selection of the reported result. Judgement about the overall risk of bias arising from the five domains was made using the published algorithm based on answers to the signalling questions within the tool. Within each domain, the risk of bias was assessed by two independent reviewers. Judgements could be 'Low', 'High' or 'Some Concerns' risk of bias.

As it is impractical and unethical to blind study participants in behavioral interventions such as caregiver interventions, we made the following modifications to the risk of bias assessment:[24,35]

1. For domain 1, allocation sequences were only considered concealed if a statement was explicitly made regarding concealment or if a computer-generated sequence was used for randomization.
2. For domain 2, we omitted item 2.3 ("Were there deviations from the intended intervention that arose because of the trial context?") from the assessment of domain 2, as all subjects were at risk to receive unintended co-interventions regardless of whether it was reported or not;
3. For domain 4, items 4.3 ("Were outcome assessors aware of the intervention received by study participants ?"), 4.4 ("Could assessment of173 the outcome have been influenced by knowledge of intervention received?") and 4.5 ("Is it likely that assessment of the outcome was influenced by knowledge of intervention received?") were omitted from final risk of bias judgements for all subjective outcomes as all studies were judged 'High' risk for this domain.

Supplementary Table 1. Study Characteristics, of Interventions Directed at Caregivers

Trial (Country)	Study Design	Patient Population and Caregiver's Relationship to Patients	Caregiver Outcomes Assessed (Instrument)	Intervention		Comparator		Results
				Description	n	Description	n	
**Arias-Rojas *et al*, 2020 (Colombia)** [41]	Single-site, individually-randomized controlled trial	Patients: stage IV cancer. Setting = inpatient. Mean age = 57.2 years.<br><br>Caregivers: family caregivers. Mean age = 43.7. Percentage female = 76%	Intervention satisfaction (31-item scale), uncertainty (26-item scale), quality of life (37-item scale)	Face-to-face nursing intervention, with educational booklet, individualized one-hour educational meeting and interactive web application	38	Group educational session	42	Uncertainty, total uncertainty, baseline to 5-7 days post intervention: Δ=-5.90 vs Δ=-2.26 (*p*=0.01)<br><br>Uncertainty, inconsistency, baseline to 5-7 days post intervention: Δ=-2.76 vs Δ=-1.24 (*p*=0.04)<br><br>Uncertainty, unpredictability, baseline to 5-7 days post intervention: Δ=-0.37 vs Δ=-0.26 (*p*>0.05)<br><br>Uncertainty, ambiguity, baseline to 5-7 days post intervention: Δ=-0.87 vs Δ=-0.57 (*p*>0.05)<br><br>Uncertainty, complexity, baseline to 5-7 days post intervention: Δ=-2.27 vs Δ=-0.26 (*p*=0.03)<br><br>Quality of life, total quality of life, baseline to 5-7 days post intervention: Δ=2.45 vs Δ=4.17 (*p*>0.05)<br><br>Quality of life, physical well-being, baseline to 5-7 days post intervention: Δ=1.48 vs Δ=0.00 (*p*>0.05)<br><br>Quality of life, psychological well-being, baseline to 5-7 days post intervention: Δ=-8.16 vs Δ=-9.55 (*p*=0.01)<br><br>Quality of life, social concerns, baseline to 5-7 days post intervention: Δ=-1.11 vs Δ=1.69 (*p*>0.05)<br><br>Quality of life, spiritual well-being, baseline to 5-7 days post intervention: Δ=-0.44 vs Δ=1.88 (*p*>0.05)

Trial (Country)	Study Design	Patient Population and Caregiver's Relationship to Patients	Caregiver Outcomes Assessed (Instrument)	Intervention		Comparator		Results
				Description	n	Description	n	
**Aubin *et al*, 2021 (Canada)** [42]	Single-site, individually-randomized controlled trial	Patients: Nonsurgical lung cancer. Setting = outpatient<br><br>Caregivers: family caregiver. Mean age = 61.8 years. Percentage female = 72%	Anxiety (HADS), caregiver preparedness (CPS), depression (HADS), distress (HADS), psychological burden (CBSEOLC), quality of life (COHQOLFCG)	Distress screening and problem assessment every 2 months, and contact with oncology nurse to review results and plan tailored problem-solving strategies	55	Usual care	54	Anxiety over time: p=0.97<br><br>Caregiver preparedness over time: p=0.61<br><br>Depression over time: p=0.86<br><br>Distress over time: p=0.90<br><br>Psychological burden over time: p=0.60<br><br>Quality of life over time: p=0.82
**Boele *et al*, 2013 (Netherlands)** [43]	Multi-site, individually-randomized controlled trial	Patients: grade III or IV high-grade gliomas. Setting = outpatient. Mean age = 52.7 years. Percentage female = 34%<br><br>Caregivers: informal caregiver, such as spouse or significant other providing > 21 hours of care per week. Mean age = 50.7 years. Percentage female = 64%	Caregiver mastery (CMS), quality of life (SF36)	Protocolized psychoeducational sessions of psychologist with caregiver, every other week for maximum of 6 one-hour sessions	31	Standard care	25	Caregiver mastery, 0 to 10 months: β=-0.369 (p=0.01)<br><br>Quality of life, mental functioning, 0 to 10 months: β=0.271 (p=0.05)
**El-Jawahri *et al*, 2020 (USA)** [44]	Single-site, individually-randomized controlled trial	Patients: autologous or allogeneic hematopoietic stem cell transplantation. Setting = outpatient<br><br>Caregiver: relative or friend who lived with patient or had in-person contact with patient at least twice per week, and identified by patient as	Anxiety (HADS), burden (CRA), coping skills (MOCS), depression (HADS), self-efficacy (CASE-T),	4 weekly and 2 semimonthly caregiver-directed coping skills intervention	45	Usual care	47	Anxiety, 0 to 2 months: d=0.96 (p<0.01)<br><br>Burden, 0 to 2 months: d=0.73 (p<0.01)<br><br>Coping skills, 0 to 2 months: d=0.98 (p<0.01)<br><br>Depression, 0 to 2 months: d=0.57 (p=0.01)<br><br>Self-efficacy, 0 to 2 months: d=0.51 (p=0.02)<br><br>Quality of life, 0 to 2 months: d=0.68 (p<0.01)

Trial (Country)	Study Design	Patient Population and Caregiver's Relationship to Patients	Caregiver Outcomes Assessed (Instrument)	Intervention		Comparator		Results
				Description	n	Description	n	
		primary caregiver. Median age = 61 years. Percentage female = 70%	quality of life (CarGOQOL)					
**Ferrell *et al*, 2019 (USA)** [45]	Single-site, individually-randomized controlled trial	Patients: stage III or IV gastrointestinal, gynecologic or genitourinary cancers. Setting = outpatient<br><br>Caregivers: family caregiver who self-identified as being financially strained by illness or treatment. Mean age = 55 years. Percentage female = 81%	Burden (CBS), distress (PDT), preparedness (CPS), quality of life (COHQOLFCG)	Four caregiver education teaching sessions delivered in-person or by phone by an advanced practice oncology nurse and a personalized self-care plan to promote caregiver well-being	117	Usual care	123	Burden, overall burden, 0 to 1 month: F=0.90 (*p*=0.34)<br><br>Burden, objective burden, 0 to 1 month: F=3.66 (*p*=0.05)<br><br>Burden, subjective stress burden, 0 to 1 month: F=0.27 (*p*=0.60)<br><br>Burden, subjective demand burden, 0 to 1 month: F=0.04 (*p*=0.83)<br><br>Distress, 0 to 1 month: F=0.53 (*p*=0.47)<br><br>Preparedness, 0 to 1 month: F=4.54 (*p*=0.03)<br><br>Quality of life, overall, 0 to 1 month: F=5.97 (*p*=0.01)<br><br>Quality of life, physical well-being, 0 to 1 month: F=1.58 (*p*=0.21)<br><br>Quality of life, psychological well-being, 0 to 1 month: F=6.21 (*p*=0.01)<br><br>Quality of life, social concerns, 0 to 1 month: F=1.61 (*p*=0.20)<br><br>Quality of life, spiritual well-being, 0 to 1 month: F=4.96 (*p*=0.02)
**Hudson *et al*, 2005 (Australia)** [46]	Multi-site, individually-randomized controlled trial	Patients: advanced cancer. Setting = home. Mean age = 69.1 years. Percentage female = 54%.	Anxiety (HADS), competence (CCS), depression (HADS),	Two educational home visits supplemented by a follow-up phone call between the two visits.	54	Usual care	52	Anxiety, at 5 weeks: 7.76 vs 8.06<br><br>Anxiety, at 8 weeks: 6.96 vs 6.76<br><br>Competence, at 5 weeks: 2.37 vs 2.40

Trial (Country)	Study Design	Patient Population and Caregiver's Relationship to Patients	Caregiver Outcomes Assessed (Instrument)	Intervention		Comparator		Results
				Description	n	Description	n	
		Caregivers: family caregiver identified by the patient. Mean age = 60.8 years. Percentage female = 65%	preparedness (PCS), rewards (RCS), self-efficacy (Zeiss Instrument)					Competence, at 8 weeks: 2.53 vs 2.47<br><br>Preparedness, at 5 weeks: 2.76 vs 2.67<br><br>Preparedness, at 8 weeks: 2.83 vs 2.59<br><br>Rewards, at 5 weeks: 3.09 vs 2.82<br><br>Rewards, at 8 weeks: 3.50 vs 3.04<br><br>Self-efficacy, problem solving, at 5 weeks: 7.37 vs 8.14<br><br>Self-efficacy, respite, at 5 weeks: 6.79 vs 6.23<br><br>Self-efficacy, rewarding interaction, at 5 weeks: 8.29 vs 8.22
**Hudson *et al*, 2013 (Australia)** [47]<br><br>**Hudson *et al*, 2015 (Australia)** [48]	Multi-site, individually-randomized controlled trial	Patients: advanced cancer, receiving home-based palliative care. Setting = home<br><br>Caregivers: family caregiver. Mean age = 59 years	Competence (CCS), family need, psychological distress (GHQ), preparedness (caregiving scale)	Three psychoeducational phone calls and one home visit in four weeks<br><br>Two psychoeducational phone calls and two home visits in four weeks	57<br><br><br><br><br>93	Usual care	148	Competence, at 5 weeks: Δ=0.30 (*p*=0.03)<br><br>Distress, at 5 weeks: Δ=0.15 (*p*>0.18)<br><br>Distress, 8 weeks after death: Δ=0.36 (*p*=0.05)<br><br>Family need, at 5 weeks: Δ=0.18 (*p*>0.07)<br><br>Preparedness, at 5 weeks: Δ=0.22 (*p*=0.08)
**Laudenslager *et al*, 2015 (USA)** [49]	Single-site, individually-randomized controlled trial	Patients: allogeneic hematopoietic stem cell transplant. Setting = mixed. Mean age = 49.5 years. Percentage female = 32%<br><br>Caregivers: caregiver. Mean age = 53.5 years. Percentage female = 76%	Anxiety (STAI), burden (CRA), depression (CESD), physical health (SF36), mental health (SF36), stress (PSS), total mood	Eight 1-on-1 educational sessions on stress and coping with social worker	74	Treatment as usual	74	Anxiety, over time: F=6.21 (*p*<0.01)<br><br>Burden, over time: F=0.92 (*p*=0.40)<br><br>Depression, over time: F=3.66 (*p*=0.03)<br><br>Physical health, over time: F=0.17<br><br>Mental health, over time: F=0.29<br><br>Total mood disturbance, over time: F=4.62 (*p*=0.01)

Trial (Country)	Study Design	Patient Population and Caregiver's Relationship to Patients	Caregiver Outcomes Assessed (Instrument)	Intervention		Comparator		Results
				Description	n	Description	n	
			disturbance (POMS-TMD), trauma (IES)					Trauma, over time: F=0.03 (*p*=0.97)
**Leow *et al*, 2015 (USA)** [50]	Multi-site, individually-randomized controlled trial	Patients: advanced cancer. Setting = mixed. Mean age = 69 years<br><br>Caregivers: family member. Mean age = 47.2 years. Percentage female = 67%	Closeness (GCS), depression (DASS), knowledge (six questions), social support (SSQ), stress (DASS), quality of life (CQOLC)	Caring for the Caregiver Programme: one-hour psychoeducational face-to-face sessions, two follow-up phone calls	38	Standard care	42	Closeness, 0 to 8 weeks: f=0.72 (*p*=0.51)<br><br>Depression, 0 to 8 weeks: f=0.72 (*p*=0.43)<br><br>Knowledge, 0 to 8 weeks: f=94.41 (*p*<0.01)<br><br>Social support (number), 0 to 8 weeks: f=1.7 (*p*=0.2)<br><br>Social support (satisfaction), 0 to 8 weeks: f=0.13 (*p*=0.83)<br><br>Stress, 0 to 8 weeks: f=1.87 (*p*=0.17)<br><br>Quality of life, 0 to 8 weeks: f=4.83 (*p*=0.01)
**McMillan *et al*, 2006 (USA)** [51]	Single-site, individually-randomized controlled trial	Patients: advanced cancer, hospice-care. Setting = home. Mean age = 70 years<br><br>Caregivers: Mean age = 60 years. Percentage female = 83%	Burden (MSAS), coping (BC) mastery (6-item scale), quality of life (CQOLC)	Three supportive visits<br><br>Three visits, to teach coping skills	109<br><br>111	Standard care	109	Burden (symptom burden), 1 to 2 weeks after intervention: β=-1.27 (*p*=0.18)<br><br>Burden (caregiving task), 1 to 2 weeks after intervention: β=-0.10 (*p*=0.06)<br><br>Coping (problem-focused), 1 to 2 weeks after intervention: β=0.05 (*p*=0.87)<br><br>Coping (emotion-focused), 1 to 2 weeks after intervention: β=0.14 (*p*=0.69)<br><br>Mastery (general), 1 to 2 weeks after intervention: β=0.33 (*p*=0.09)<br><br>Mastery (caregiver task), 1 to 2 weeks after intervention: β=0.03 (*p*=0.51)<br><br>Quality of life, 1 to 2 week after intervention: β=-2.03 (*p*=0.16)

Trial (Country)	Study Design	Patient Population and Caregiver's Relationship to Patients	Caregiver Outcomes Assessed (Instrument)	Intervention		Comparator		Results
				**Description**	**n**	**Description**	**n**	
**Mitchell *et al*, 2013 (Australia)** [52]	Multi-site, individually-randomized controlled trial	Patients: locally invasive or metastatic cancer. Setting = mixed.<br><br>Caregivers: not defined. Mean age = 57.4 years. Percentage female = 56%	Anxiety (HADS), depression (HADS), unmet needs (SCNS-P&C), quality of life (SF12)	Carer-practitioner consultations directed by self-completed checklist of needs	194	Usual care	198	Anxiety, at 1 month: d=0.05 (*p*=0.886)<br><br>Anxiety, at 3 months: d = 0.33 (*p*=0.437)<br><br>Anxiety, at 6 months: d=0.82 (*p*=0.109)<br><br>Depression, at 1 month: d=0.22 (*p*=0.473)<br><br>Depression, at 3 months: d=0.21 (*p*=0.624)<br><br>Depression, at 6 months: d=0.73 (*p*=0.154)<br><br>Total number of unmet needs, at 1 month: d=0.18 (*p*=0.772)<br><br>Total number of unmet needs, at 3 months: d=0.49 (*p*=0.441)<br><br>Total number of unmet needs, at 6 months: d=0.16 (*p*=0.841)<br><br>Quality of life, physical component, at 1 month: d=0.61 (*p*=0.482)<br><br>Quality of life, physical component, at 3 months: d=0.85 (*p*=0.362)<br><br>Quality of life, physical component, at 6 months: d=0.14 (*p*=0.902)<br><br>Quality of life, mental component, at 1 month: d=0.45 (*p*=0.662)<br><br>Quality of life, mental component, at 3 months: d=1.49 (*p*=0.178)<br><br>Quality of life, mental component, at 6 months: d=0.13 (*p*=0.924)
**Pensak *et al*, 2020 (USA)** [53]	Single-site, individualy-	Patients: advanced cancer. Setting = outpatient	Ability (MOCS), anxiety	12-week self-administered educational	26	Treatment as usual	30	Ability, over time: F=6.16 (*p*=0.02)<br><br>Anxiety, over time: F=0.27 (*p*=0.61)

Trial (Country)	Study Design	Patient Population and Caregiver's Relationship to Patients	Caregiver Outcomes Assessed (Instrument)	Intervention		Comparator		Results
				Description	n	Description	n	
	randomized controlled trial	Caregivers: primary caregiver. Mean age = 54.3 years. Percentage female = 75%	(HADS), depression (CESD), female sexual function (FSFI), male sexual health (MSHQ), stress (PSS)	intervention, of 10 prerecorded sessions of less than 20 minutes				Depression, over time: F=0.10 (p=0.75)<br><br>Female sexual function, over time: F=5.07 (p=0.03)<br><br>Male sexual health, over time: F=1.06 (p=0.33)<br><br>Stress, over time: F=3.91 (p=0.05)
**Walsh *et al*, 2007 (United Kingdom)** [54]	Multi-site, individually-randomized controlled trial	Patients: advanced cancer, receiving specialist palliative care. Setting = outpatient<br><br>Caregivers: identified by patient. Mean age = 56.3 years. Percentage female = 79%	Bereavement (CBereavementI), strain (CSI), quality of life (CQOLC)	Six visits with community nursing and social work over 6 weeks	137	Specialist palliative care	134	Bereavement, 4 months after death: t=0.91 (p=0.37)<br><br>Strain, at 4 weeks: 27.7 vs 27.8<br><br>Strain, at 9 weeks: 26.7 vs 25.1<br><br>Strain, at 12 weeks: 27.2 vs 27.3<br><br>Quality of life, at 4 weeks: 72.8 vs 66.4<br><br>Quality of life, at 9 weeks: 69.6 vs 63.9<br><br>Quality of life, at 12 weeks: 65.2 vs 62.2
**Washington *et al*, 2018 (USA)** [55]	Single-site, individually-randomized controlled trial	Patient: palliative cancer. Setting = outpatient. Mean age = 60.4 years. Percentage female = 53%<br><br>Caregivers: family caregiver. Mean age = 51.5 years. Percentage female = 69%	Anxiety (GAD-7), depression (PHQ-9), quality of life (CQLI-R)	Three problem-solving intervention sessions, each 1 week apart	42	Usual care	41	Anxiety: β=-2.76 vs 16.36<br><br>Depression: β=-2.68 vs 12.80<br><br>Quality of life: β=2.77 vs -5.29
**Younis *et al*, 2019 (Jordan)** [56]	Single-site, individually-randomized controlled trial	Patient: cancers receiving palliative care. Setting = outpatient<br><br>Caregivers: non-professional caregiver	Quality of life (QOLLTI-F)	Formal teaching in structured environment, and home visits to provide teaching and training	47	Control group, no intervention	45	Quality of life: d=13.62 (p<0.001)

Trial (Country)	Study Design	Patient Population and Caregiver's Relationship to Patients	Caregiver Outcomes Assessed (Instrument)	Intervention		Comparator		Results
				Description	n	Description	n	
				Home visits only to provide teaching and training	45			Quality of life: d=-1.09 (*p*=1.00)
**Yun *et al*, 2011 (Korea)** [57]	Multi-site, individually-randomized controlled trial	Patient: terminal cancer. Setting = outpatient. Mean age = 61.9 years. Percentage female = 43%<br><br>Caregivers: family caregiver. Mean age = 45.6 years. Percentage female = 66%	Anxiety (HADS), decisional conflict (DCS), depression (HADS), quality of life (CQOLC)	Educational video and companion workbook	216	Educational video and workbook on pain control	228	Anxiety: p=0.51<br><br>Decisional conflict: p=0.04<br><br>Depression: p<0.01<br><br>Quality of life: p=0.99

Legend:

BC – Brief Cope
CarGOQOL – Caregiver Oncology Quality of Life Questionnaire
CASE-T – Cancer Self-Efficacy Scale – Transplant
CBereavementI – Core Bereavement Items
CBS – Care-Giving Burden Scale
CBSEOLC – Caregiver's Burden Scale in End-of-Life Care
CCS – Caregiver Competence Scale
CESD – Center for Epidemiological Studies – Depression Scale
CMS – Caregiver Mastery Scale
COHQOLFCG – City of Hope Quality of Life Family Caregiver Version
CPS – Caregiving Preparedness Scale
CQLI-R – Caregiver Quality of Life Index – Revised
CQOLC – Caregiver Quality of Life Index – Cancer
CRA – Caregivers Reaction Assessment Scale
CSI – Caregiver Strain Index
DASS – Depression Anxiety Stress Scales
DCS – Decision Conflict Scale
FSFI – Female Sexual Function Index
GAD-7 – Generalized Anxiety Disorder 7-item Scale
GCS - General Closeness Scale
GHQ – General Health Questionnaire

HADS – Hospital Anxiety and Depression Scale
IES – Impact of Events Scale
MOCS – Measure of Current Status
MSAS – Memorial Symptom Assessment Scale
MSHQ – Male Sexual Health Questionnaire
PCS – Preparedness for Caregiving Scale
PDT – Preparedness Scale
PHQ-9 – Patient Health Questionnaire 9-item Scale
POMS-TMD – Profile of Mood States, Total Mood Disturbance
PSS – Perceived Stress Scale
QOLLTI-F – Quality of Life in Life-Threatening Illness
RCS – Rewards of Caregiving Scale
SCNS-P&C – Supportive Care Needs Survey – Partners and Carers
SF12 – 12-Item Short-Form Health Survey
SF36 – 36-Item Short-Form Health Survey
SSQ – Social Support Questionnaire
STAI – State-Trait Anxiety Inventory

Supplementary Table 2. Study Characteristics, of Interventions Directed at Patient-Caregiver Dyad

Trial (Country)	Study Design	Patient Population and Caregiver's Relationship to Patients	Caregiver Outcomes Assessed (Instrument)	Intervention		Comparator		Results
				Description	n	Description	n	
**Ammari *et al*, 2018 (Denmark)** [58]	Multi-site, individually-randomized controlled trial	Patients: stage III-IV advanced solid cancer. Setting = mixed. Mean age = 67.4 years. Percentage female = 32%<br><br>Caregivers: closest relative of patient. Mean age = 62.1 years. Percentage female = 75%	Anxiety (HADS), depression (HADS), quality of life (SF36)	Six home visits by a nurse, over 3 weeks, of family coping oriented palliative homecare for patients and their closest relative	28	Usual treatment and care, including conventional community based homecare if necessary	29	Anxiety, at 16 weeks: β=-0.51 (*p*=0.64)<br><br>Anxiety, at 24 weeks: β=-0.51 (*p*=0.64)<br><br>Depression, at 16 weeks: β=-1.24 (*p*=0.09)<br><br>Depression, at 24 weeks: β=-0.01 (*p*=0.99)<br><br>Quality of life, mental component, at 16 weeks: β=1.72 (*p*=0.47)<br><br>Quality of life, mental component, at 24 weeks: β=-6.12 (*p*=0.12)<br><br>Quality of life, physical component, at 16 weeks: β=-1.79 (*p*=0.64)<br><br>Quality of life, physical component, at 24 weeks: β=2.05 (*p*=0.40)
**Badr *et al*, 2015 (USA)** [59]	Single-site, individually-randomized controlled trial	Patients: advanced lung cancer. Setting = outpatient. Mean age = 68.2 years. Percentage female = 74%<br><br>Caregivers: spouse/partner or other close family member. Mean age = 51.1 years. Percentage female = 69%	Anxiety (PROMIS SF Anxiety), autonomy (Pierre instrument), burden (Zarit burden interview), competence (38-item questionnaire), depression (PROMIS SF Depression), relatedness (4-	Separate standardized tailored manuals, divided into 6 sections, for patients and caregivers, who participated together in 6 weekly 60-minute telephone counseling sessions	20	Standard oncologic care and primary palliative care for the patient	19	Anxiety, at 8 weeks: F=34.34 (*p*<0.01)<br><br>Autonomy, at 8 weeks: F=21.46 (*p*<0.01)<br><br>Burden, at 8 weeks: F=21.46 (*p*<0.01)<br><br>Competence, at 8 weeks: F=132.34 (*p*<0.01)<br><br>Depression, at 8 weeks: F=72.13 (*p*<0.01)<br><br>Relatedness, at 8 weeks: F=63.66 (*p*<0.01)

Trial (Country)	Study Design	Patient Population and Caregiver's Relationship to Patients	Caregiver Outcomes Assessed (Instrument)	Intervention		Comparator		Results
				Description	n	Description	n	
			item questionnaire)					
**Chih *et al*, 2013 (USA)** [60]<br><br>**DuBenske *et al*, 2014 (USA)** [61]<br><br>**Namkoong *et al*, 2012 (USA)** [62]	Multi-site, individually-randomized controlled trial	Patients: stage IIIA, IIIB or IV non-small cell lung cancer. Setting = outpatient.<br><br>Caregivers: caregivers. Mean age = 55.6 years. Percentage female = 68%	Burden (CQOLC), disruptiveness (CQOLC), negative mood* (SV-POMS), physical burden (CBI), preparedness (FCI)	Comprehensive Health Enhancement Support System (CHESS) website with electronic patient-reported outcome system (Clinician Report) to deliver needs and patient symptoms to clinician	118<br><br><br><br><br><br><br><br><br><br><br><br><br><br><br><br>144	CHESS without Clinician Report<br><br><br><br><br><br><br><br><br><br><br><br><br><br><br><br>Standard care plus laptop computer with Internet access and list of lung cancer and palliative care websites	117<br><br><br><br><br><br><br><br><br><br><br><br><br><br><br><br>141	Negative mood, at 6 months: 0.62 vs 0.88 (*p*<0.01)<br><br>Negative mood, at 12 months: 0.61 vs 0.93 (*p*<0.01)<br><br>Physical burden, at 6 months: 1.22 vs 1.22 (*p*=0.98)<br><br>Physical burden, at 12 months: 1.27 vs 1.22 (*p*=0.73)<br><br>Preparedness, at 6 months: 2.91 vs 2.83 (*p*=0.42)<br><br>Preparedness, at 12 months: 2.82 vs 2.79 (*p*=0.78)<br><br>Bonding, at 6 months: β=0.17 (*p*<0.05)<br><br>Burden, 0 to 8 months: d=0.22 (*p*=0.05)<br><br>Coping (active behavior), at 6 months: β=-0.02 (*p*>0.05)<br><br>Coping (positive reframing), at 6 months: β=-0.15 (*p*>0.05)<br><br>Coping (instrumental support), at 6 months: β=-0.05 (*p*>0.05)<br><br>Disruptiveness, 0 to 8 months: d=0.21 (*p*=0.07)<br><br>Negative mood, 0 to 8 months: d=0.21 (*p*=0.05)
**Clark *et al*, 2013 (USA)** [63]	Single-site, individually-randomized controlled trial	Patients: advanced cancer. Setting = outpatient. Mean age =	Mood (POMB), Quality of life	Six structured 90-minute multidisciplinary sessions, where	65	Standard medical care	66	Mood, at 4 weeks: Δ=0.32 (*p*=0.02)<br><br>Mood, at 27 weeks: Δ=0.27 (*p*=0.36)

Trial (Country)	Study Design	Patient Population and Caregiver's Relationship to Patients	Caregiver Outcomes Assessed (Instrument)	Intervention		Comparator		Results
				Description	n	Description	n	
**Lapid *et al*, 2016 (USA)** [64]		59.3 years. Percentage female = 34%<br><br>Caregivers: caregiver.	(CQOLC, LASA)	caregivers invited for sessions 1, 3, 4 and 6				Mood, tension/anxiety, at 4 weeks: Δ=0.18 (*p*=0.61)<br><br>Mood, tension/anxiety, at 27 weeks: Δ=0.16 (*p*=0.63)<br><br>Mood, vigor/activity, at 4 weeks: Δ=0.48 (*p*=0.02)<br><br>Mood, vigor/activity, at 27 weeks: Δ=0.44 (*p*=0.07)<br><br>Mood, fatigue/inertia, at 4 weeks: Δ=0.45 (*p*=0.02)<br><br>Mood, fatigue/inertia, at 27 weeks: Δ=0.57 (*p*=0.01)<br><br>Mood, depression/dejection, at 4 weeks: Δ=0.18 (*p*=0.11)<br><br>Mood, depression/dejection, at 27 weeks: Δ=0.11 (*p*=0.73)<br><br>Mood, confusion/bewilderment, at 4 weeks: Δ=0.11 (*p*=0.44)<br><br>Mood, confusion/bewilderment, at 27 weeks: Δ=0.18 (*p*=0.76)<br><br>Mood, anger/hostility, at 4 weeks: Δ=0.15 (*p*=0.89)<br><br>Mood, anger/hostility, at 27 weeks: Δ=0.04 (*p*=0.85)<br><br>Quality of life (LASA), at 4 weeks: Δ=0.18 (*p*=0.26)

Trial (Country)	Study Design	Patient Population and Caregiver's Relationship to Patients	Caregiver Outcomes Assessed (Instrument)	Intervention		Comparator		Results
				Description	n	Description	n	
								Quality of life (LASA), at 27 weeks: Δ=0.14 (*p*=0.65)<br><br>Quality of life (LASA), mental well-being, at 4 weeks: Δ=0.16 (*p*=0.24)<br><br>Quality of life (LASA), mental well-being, at 27 weeks: Δ=0.17 (*p*=0.42)<br><br>Quality of life (LASA), physical well-being, at 4 weeks: Δ=0.30 (*p*=0.08)<br><br>Quality of life (LASA), physical well-being, at 27 weeks: Δ=0.30 (*p*=0.10)<br><br>Quality of life (LASA), emotional well-being, at 4 weeks: Δ=0.02 (*p*=0.87)<br><br>Quality of life (LASA), emotional well-being, at 27 weeks: Δ=0.16 (*p*=0.61)<br><br>Quality of life (LASA), social activity, at 4 weeks: Δ=0.03 (*p*=0.82)<br><br>Quality of life (LASA), social activity, at 27 weeks: Δ=0.17 (*p*=0.40)<br><br>Quality of life (LASA), spiritual well-being, at 4 weeks: Δ=0.29 (*p*=0.05)<br><br>Quality of life (LASA), spiritual well-being, at 27 weeks: Δ=0.13 (*p*=0.46)<br><br>Quality of life (LASA), fatigue, at 4 weeks: Δ=0.03 (*p*=0.93)<br><br>Quality of life (LASA), fatigue, at 27 weeks: Δ=0.04 (*p*=0.76)

Trial (Country)	Study Design	Patient Population and Caregiver's Relationship to Patients	Caregiver Outcomes Assessed (Instrument)	Intervention		Comparator		Results
				Description	n	Description	n	
								Quality of life (LASA), social support, at 4 weeks: Δ=0.13 (*p*=0.76)<br><br>Quality of life (LASA), social support, at 27 weeks: Δ=0.17 (*p*=0.36)<br><br>Quality of life (LASA), financial well-being, at 4 weeks: Δ=0.06 (*p*=0.27)<br><br>Quality of life (LASA), financial well-being, at 27 weeks: Δ=0.40 (*p*=0.05)<br><br>Quality of life (LASA), legal concerns, at 4 weeks: Δ=0.07 (*p*=0.58)<br><br>Quality of life (LASA), legal concerns, at 27 weeks: Δ=0.25 (*p*=0.33)<br><br>Quality of life (CQOLC), at 4 weeks: Δ=0.20 (*p*=0.32)<br><br>Quality of life (CQOLC), at 27 weeks: Δ=0.20 (*p*=0.38)<br><br>Quality of life (CQOLC), burden, at 4 weeks: Δ=0.04 (*p*=0.95)<br><br>Quality of life (CQOLC), burden, at 27 weeks: Δ=0.01 (*p*=0.93)<br><br>Quality of life (CQOLC), disruptiveness, at 4 weeks: Δ=0.16 (*p*=0.28)<br><br>Quality of life (CQOLC), disruptiveness, at 27 weeks: Δ=0.38 (*p*=0.05)<br><br>Quality of life (CQOLC), adaptation, at 4 weeks: Δ=0.48 (*p*=0.01)

Trial (Country)	Study Design	Patient Population and Caregiver's Relationship to Patients	Caregiver Outcomes Assessed (Instrument)	Intervention		Comparator		Results
				Description	n	Description	n	
								Quality of life (CQOLC), adaptation, at 27 weeks: Δ=0.35 (*p*=0.07)<br><br>Quality of life (CQOLC), financial concerns, at 4 weeks: Δ=0.15 (*p*=0.62)<br><br>Quality of life (CQOLC), financial concerns, at 27 weeks: Δ=0.46 (*p*=0.02)
**Dionne-Odom *et al*, 2015 (USA)** [65]<br><br>**Dionne-Odom *et al*, 2016 (USA)** [66]	Multi-site, individually-randomized controlled trial	Patients: advanced stage recurrent or progressive metastatic cancer. Setting = outpatient.<br><br>Caregivers: knows the patient well and involved in medical care. Mean age = 59.5 years. Percentage female = 79%	Burden (MBCB), depression (CESD), grief (PG13), quality of life (CQOLC)	Educate, Nurture, Advise Before Life Ends (ENABLE) intervention plus three structured weekly caregiver telephone coaching sessions with an advanced practice palliative care nurse, monthly follow-up and a bereavement call	61	ENABLE intervention, 3 months after random assignment	61	Burden, objective burden, 0 to 3 months: d=0.09 (*p*=0.62)<br><br>Burden, objective burden, 0 to 36 weeks before death: d=-0.18 (*p*=0.27)<br><br>Burden, stress burden, 0 to 3 months: d=-0.21 (*p*=0.27)<br><br>Burden, stress burden, 0 to 36 weeks before death: d=-0.44 (*p*=0.01)<br><br>Burden, demand burden, 0 to 3 months: d=0 (*p*=0.99)<br><br>Burden, stress burden, 0 to 36 weeks before death: d=-0.23 (*p*=0.22)<br><br>Depression, 0 to 3 months: d=-0.32 (*p*=0.02)<br><br>Depression, 0 to 36 weeks before death: d=-0.39 (*p*=0.02)<br><br>Depression, after death: d=-0.34 (*p*=0.39)<br><br>Grief, after death: d=-0.35 (*p*=0.21)<br><br>Quality of life, 0 to 3 months: d=-0.31 (*p*=0.37)<br><br>Quality of life, 0 to 36 weeks before death: d=-0.3 (*p*=0.07)

Trial (Country)	Study Design	Patient Population and Caregiver's Relationship to Patients	Caregiver Outcomes Assessed (Instrument)	Intervention		Comparator		Results
				Description	n	Description	n	
**Keefe *et al*, 2005 (USA) [67]**	Multi-site, individually-randomized controlled trial	Patient: advanced cancer. Setting = outpatient. Mean age = 60.5 years. Percentage male = 56%<br><br>Caregivers: spouse. Mean age = 58.5 years. Percentage female = 62%	Mood (POMB), self-efficacy (CPSS), strain (CSI)	Three face-to-face sessions in patient's home that integrated education about cancer pain with training of patients and caregivers in pain management strategies	41	Routine care through medical outpatient or hospice program	37	Mood (negative mood), post-treatment: 0.89 vs 1.07 (*p*=0.24)<br><br>Mood (positive mood), post-treatment: 1.77 vs 1.94 (*p*=0.36)<br><br>Self-efficacy, pain, post-treatment: 62.64 vs 39.59 (*p*<0.01)<br><br>Self-efficacy, physical activities, post-treatment: 62.70 vs 58.32<br><br>Self-efficacy, other symptoms, post-treatment: 68.75 vs 52.46 (*p*=0.01)<br><br>Strain, post-treatment: 6.39 vs 7.17 (*p*=0.06)
**Kubo *et al*, 2020 (USA) [68]**	Multi-site, cluster-randomized controlled trial	Patients: metastatic solid malignancies or hematological cancers. Setting = outpatient. Mean age = 66.4 years. Percentage female = 70%<br><br>Caregivers: primary informal caregivers. Mean age = 62.4 years. Percentage female = 79%	Distress (NCCNDT), mindfulness (FFMQ-SF)	6-week mobile-based mindfulness program or 6-week webinar-based mindfulness program	22	Waitlist control arm, receiving usual care	17	Distress, over time: *p*>0.05<br><br>Mindfulness, over time: *p*>0.05
**Li *et al*, 2019 (China) [69]**	Single-site, individually-randomized controlled trial	Patients: stage III-IV non-small cell lung cancer. Setting = outpatient. Mean age = 55 years. Percentage female = 70%<br><br>Caregivers: spouse or adult child living with patient. Mean age = 50	Family relationships (FES), quality of life (CQOLC)	Six 45-minute sessions of multidisciplinary components including physical therapy, coping and communication strategies, and education about cancer symptoms,	80	Control group	79	Family relationships, cohesion, at 8 weeks: Δ=0.07 (*p*=0.02)<br><br>Family relationships, conflict, at 8 weeks: Δ=1.08 (*p*=0.03)<br><br>Family relationships, expressiveness, at 8 weeks: Δ=0.03 (*p*=0.06)<br><br>Quality of life, burden, at 8 weeks: Δ=1.5 (*p*=0.08)

Trial (Country)	Study Design	Patient Population and Caregiver's Relationship to Patients	Caregiver Outcomes Assessed (Instrument)	Intervention		Comparator		Results
				Description	n	Description	n	
		years. Percentage female = 86%		physical and mental health, and treatment, over 8 weeks				Quality of life, disruptiveness, at 8 weeks: Δ=3.4 (*p*=0.06)<br><br>Quality of life, adaptation, at 8 weeks: Δ=6.7 (*p*=0.04)<br><br>Quality of life, financial, at 8 weeks: Δ=5.9 (*p*=0.05)
**McLean *et al*, 2013 (Canada)** [70]	Single-site, individually-randomized controlled trial	Patients: metastatic cancer. Setting = outpatient. Mean age = 50.7 years. Percentage female = 55%<br><br>Caregivers: Romantic partner. Mean age = 49.8 years. Percentage female = 45%	Burden (CBurdenI), depression (BDI), hopelessness (BHS), marital functioning (RDAS)	Eight-session one-hour emotionally-focused therapy intervention adapted for couples in the advanced cancer setting	22	Standard care	20	Burden, after intervention: F=49.69 (*p*<0.01)<br><br>Burden, 3 months after intervention: F=12.00 (*p*<0.01)<br><br>Depression, after intervention: F=30.43 (*p*<0.01)<br><br>Depression, 3 months after intervention: F=33.62 (*p*<0.01)<br><br>Hopelessness, after intervention: F=38.87 (*p*<0.01)<br><br>Hopelessness, 3 months after intervention: F=78.35 (*p*<0.01)<br><br>Marital functioning, after intervention: F=35.38 (*p*<0.01)<br><br>Marital functioning, 3 months after intervention: F=91.08 (*p*<0.01)
**Meyers *et al*, 2011 (USA)** [71]	Multi-site, individually-randomized controlled trial	Patients: advanced cancer. Setting = outpatient. Mean age = 61.5 years. Percentage female = 56%<br><br>Caregivers: adult regularly involved with patient and their care. Mean age = 61.4 years.	Social problem solving (SPSI), quality of life (COHQOL)	Three sessions of the COPE cognitive-behavioral problem-solving educational intervention during one month	324	Standard care	117	Social problem solving (avoidance): β=0.38 (*p*=0.12)<br><br>Social problem solving (impulsivity): β=-0.31 (*p*=0.22)<br><br>Social problem solving (negative approaches): β=0.12 (*p*=0.62)

Trial (Country)	Study Design	Patient Population and Caregiver's Relationship to Patients	Caregiver Outcomes Assessed (Instrument)	Intervention		Comparator		Results
				Description	n	Description	n	
		Percentage female = 31%						Social problem solving (positive approaches): β=-0.47 (*p*=0.17)<br><br>Social problem solving (rational approaches): β=-0.30 (*p*=0.33)<br><br>Quality of life (physical): β=0.09 (*p*=0.61)<br><br>Quality of life (psychological): β=0.59 (*p*=0.16)<br><br>Quality of life (social): β=0.46 (*p*=0.09)<br><br>Quality of life (spiritual): β=0.76 (*p*<0.01)
**Milbury *et al*, 2020 (USA)** [72]	Single-site, individually-randomized controlled trial	Patients: stage IV non small cell lung cancer. Setting = outpatient. Mean age = 64.6 years. Percentage female = 51%<br><br>Caregivers: spouse. Mean age = 63.6 years. Percentage female = 52%	Depression (CESD), spiritual well-being (FACT-SWB), stress (IES)	Four weekly 60-minute couple-based meditation sessions via videoconference with psychological counselor intern	50	Usual care	25	Depression, over time: F=3.70 (*p*<0.05)<br><br>Spiritual well-being, over time: F=1.79 (*p*=0.17)<br><br>Stress, over time: F=0.32 (*p*=0.73)
**Mosher *et al*, 2018 (USA)** [73]	Multi-site, individually-randomized controlled trial	Patients: stage IV GI cancer. Setting = outpatient.<br><br>Caregivers: family member. Percentage female = 66%	Anxiety (PROMIS SF Anxiety), burden (Zarit interview), depression (PROMIS SF Depression), distress (Distress Thermometer), emotional support (PROMIS SF Emotional Support),	Five sessions, with therapist, of Peer Helping intervention (contributing to handouts on coping skills for other patients and caregivers coping with cancer) in addition to Coping Skills intervention (discussing own experiences related to various	25	Four sessions with therapist of Coping Skills intervention alone	25	Anxiety, 0 to 5 weeks after intervention: F=1.88 (*p*=0.16)<br><br>Burden, 0 to 5 weeks after intervention: F=4.74 (*p*=0.01)<br><br>Depression, 0 to 5 weeks after intervention: F=2.55 (*p*=0.09)<br><br>Distress, 0 to 5 weeks after intervention: F=2.57 (*p*=0.08)<br><br>Emotional support, 0 to 5 weeks after intervention: F=1.64 (*p*=0.20)

Trial (Country)	Study Design	Patient Population and Caregiver's Relationship to Patients	Caregiver Outcomes Assessed (Instrument)	Intervention		Comparator		Results
				Description	n	Description	n	
			fatigue (PROMIS SF Fatigue), self-efficacy (MOCS), sense of meaning in life and peace (FACIT-Sp)	quality of life issues)				Fatigue, 0 to 5 weeks after intervention: F=7.08 (*p*<0.01)<br><br>Self-efficacy, 0 to 5 weeks after intervention: F=1.31 (*p*=0.28)<br><br>Sense of meaning in life and peace, 0 to 5 weeks after intervention: F=3.37 (*p*=0.04)
**Northouse *et al*, 2005 (USA)** [74]	Multi-site, individually-randomized trial	Patients: advanced breast cancer. Setting = outpatient. Mean age = 54 years.<br><br>Caregivers: family member or significant other identified as primary source of emotional and physical support. Mean age = 52 years.	Appraisal (ACS), coping (BC), hopelessness (BHS), quality of life (FACT-G) uncertainty (MUIS)	FOCUS Program: Family involvement, Optimistic attitude, Coping effectiveness, Uncertainty reduction and Symptom management. Three 1.5 hour home visits with patient and caregiver, and two 30-minute phone calls	94	Usual care	88	Appraisal, from 0-3 months: F=3.90 (*p*=0.04)<br><br>Appraisal, from 3-6 months: F=0.80 (*p*=0.37)<br><br>Coping (active coping), from 0-3 months: F=0.19 (*p*=0.67)<br><br>Coping (active coping), from 3-6 months: F=1.03 (*p*=0.31)<br><br>Coping (avoidant coping), from 0-3 months: F=0.63 (*p*=0.43)<br><br>Coping (avoidant coping), from 3-6 months: F=0.82 (*p*=0.37)<br><br>Hopelessness, from 0-3 months: F=0.07 (*p*=0.80)<br><br>Hopelessness, from 3-6 months: F=1.48 (*p*=0.23)<br><br>Quality of life (mental), from 0-3 months: F=0.06 (*p*=0.81)<br><br>Quality of life (mental), from 3-6 months: F=0.32 (*p*=0.57)<br><br>Quality of life (physical), from 0-3 months: F=0.01 (*p*=0.91)<br><br>Quality of life (physical), from 3-6 months: F=0.51 (*p*=0.48)

Trial (Country)	Study Design	Patient Population and Caregiver's Relationship to Patients	Caregiver Outcomes Assessed (Instrument)	Intervention		Comparator		Results
				Description	n	Description	n	
								Uncertainty, from 0-3 months: F=1.13 (*p*=0.07)<br><br>Uncertainty, from 3-6 months: F=1.57 (*p*=0.21)
**Northouse *et al*, 2013 (USA)** [75]	Multi-site, individually-randomized controlled trial	Patients: advanced breast, colorectal, lung or prostate cancer. Setting = outpatient. Mean age: 60.5 years. Percentage female = 61%<br><br>Caregivers: primary caregiver identified by patient. Mean age 56.7 years. Percentage female = 56%	Appraisals of illness (ACS), communication (LMSS), coping strategies (BC), dyadic support (SSQ), healthy behaviors (scale), hopelessness (BHS), uncertainty (MUIS), risk for distress, self-efficacy (LCSS), quality of life (FACT)	Brief FOCUS: three contacts (two 90-min house visits and one 30-min phone sessions)<br><br>Extensive FOCUS: six contacts (four 90-min house visits and two 30-min phone sessions)	159<br><br>162	Usual care	163	Appraisal, hopelessness and uncertainty, 0 to 6 months: F=0.99 (*p*=0.46)<br><br>Coping strategies, 0 to 6 months: F=2.15 (*p*=0.01)<br><br>Communication and dyadic support, 0 to 6 months: F=1.04 (*p*=0.41)<br><br>Self-efficacy, 0 to 6 months: F=2.84 (*p*=0.02)<br><br>Quality of life, social domain, 0 to 6 months: F:4.28 (*p*<0.01)<br><br>Quality of life, emotional domain, 0 to 6 months: F=0.80 (*p*=0.52)<br><br>Quality of life, functional domain, 0 to 6 months: F=0.35 (*p*=0.84)<br><br>Quality of life, physical domain, 0 to 6 months: F=1.16 (*p*=0.33)
**Porter *et al*, 2017 (USA)** [76]	Single-site, individually-randomized controlled trial	Patients: stage 3 or 4 gastrointestinal cancer. Setting = outpatient. Mean age = 54.7 years. Percentage female = 31%<br><br>Caregiver: spouse. Mean age = 52.3 years. Percentage female = 69%	Acceptability (CSQ8), communication (MSI), distress (IES), intimacy (MSIS), psychological growth (PTGI), relationship satisfaction (RDAS), self-efficacy (SSS)	Couples Communication Skills Training: systematically trained couples in two communication skills to help patients and their partners (i) disclose their thoughts and feelings related to the cancer experience to each	15	Health Lifestyle Information: provided health information relevant to cancer in a supportive environment.	17	Acceptability, after intervention: 3.43 vs 3.18<br><br>Communication, affective communication, after intervention: ES=-0.14<br><br>Communication, problem solving communication, after intervention: ES=-0.07<br><br>Distress, after intervention: ES=-0.14<br><br>Intimacy, after intervention: ES=-0.07<br><br>Psychological growth, after intervention: ES=0.18

Trial (Country)	Study Design	Patient Population and Caregiver's Relationship to Patients	Caregiver Outcomes Assessed (Instrument)	Intervention		Comparator		Results
				Description	n	Description	n	
				other and (ii) make decisions about cancer-related issues.				Relationship satisfaction, after intervention: ES=0.34<br><br>Self-efficacy, after intervention: ES=-0.25
**Sherwood *et al*, 2012 (USA)** [77]	Multi-site, individually-randomized controlled trial	Patients: stage III or IV solid tumor. Setting = outpatient<br><br>Caregivers: family caregiver. Mean age = 55.0 years. Percentage female = 60%	Burden (CRS), communication (ENRICH), depression (CESD), mastery (7-item scale), symptom involvement	Telephone calls (3 for caregivers, 6 for patients) by a Master's prepared nurse with experience in oncology, with problem-solving techniques for symptom management	112	Attention control: same number of telephone calls by a research staff member without providing problem-solving techniques	113	Burden: β=0.02 (p=0.62)<br><br>Communication: β=-0.60 (p=0.07)<br><br>Depression: β=1.34 (p<0.01)<br><br>Mastery: β=-0.90 (p<0.01)<br><br>Symptom involvement: β=-0.90 (p<0.01)
**von Heymann-Horan *et al*, 2018 (Denmark)** [78]<br><br>**von Heymann-Horan *et al*, 2019 (Denmark)** [79]	Single-site, individually-randomized controlled trial	Patients: incurable cancer. Setting = home. Mean age = 66 years. Percentage female = 54%<br><br>Caregivers: caregiver. Mean age = 45 years. Percentage female = 62%	Anxiety (SC92), common coping (DCI), communication (DCI), depression (SC92)	Home-based specialized palliative care and dyadic psychological support (two 90-minute sessions, and as-needed monthly follow-up)	134	Usual care	115	Common coping: Δ=0.40<br><br>Communication: Δ=0.66
**Wang *et al*, 2021 (China)** [80]	Single-site, individually-randomized controlled trial	Patients: hematologic cancers. Setting = inpatient. Mean age = 49.4 years. Percentage female = 47%<br><br>Caregivers: patient's family members or close relatives, undertaking primary responsibility of caring for patient. Mean age = 38.3 years. Percentage female = 44%	Anxiety (SA), depression (SDS), family adaptability and cohesion (FACESII)	Four sessions of dyadic dignity therapy delivered by a therapist to the patient and family caregiver	27	Routine care	26	Anxiety, over time: F=5.88 (p<0.01)<br><br>Depression, over time: F=2.58 (p=0.06)<br><br>Family adaptability, over time: F=2.23 (p=0.12)<br><br>Family cohesion, over time: F=3.13 (p=0.04)

Trial (Country)	Study Design	Patient Population and Caregiver's Relationship to Patients	Caregiver Outcomes Assessed (Instrument)	Intervention		Comparator		Results
				Description	n	Description	n	
**Yanwei *et al*, 2018 (China) [81]**	Single-site, individually-randomized controlled trial	Patients: stage IV non-small cell lung cancer. Setting = outpatient. Percentage female = 62%<br><br>Caregivers: Family. Percentage female = 47%	Anxiety (HADS), depression (HADS), family relationships (FES), quality of life (CQOLC)	At least four 30-minute sessions of multidisciplinary components over 8 weeks, including physical therapy, coping and communication strategies, and education about cancer symptoms, physical and mental health, and cancer treatment	64	Usual care	62	Anxiety, at 12 weeks: 3.1 vs 0.9 ($p<0.05$)<br><br>Depression, at 12 weeks: 2.9 vs 0.5 ($p<0.05$)<br><br>Family relationships, cohesion, at 12 weeks: 0.6 vs 0.7 ($p>0.05$)<br><br>Family relationships, conflict, at 12 weeks: 1.7 vs 1.1 ($p>0.05$)<br><br>Family relationships, expressiveness, at 12 weeks: 0.7 vs 1.0 ($p>0.05$)<br><br>Quality of life, at 12 weeks: 3.9 vs 1.7 ($p>0.05$)

Legend:

ACS – Appraisal of Caregiving Scale
BC – Brief Cope
BDI – Beck Depression Inventory
BHS – Beck Hopelessness Scale
CBI – Caregiver Burden Inventory
CBurdenI – Caregiver Burden Inventory
CESD – Center for Epidemiologic Studies – Depression
CHESS - Comprehensive Health Enhancement Support System
COHQOL – City of Hope Quality of Life Instrument
CPSS – Chronic Pain Self-Efficacy Scale
CQOLC – Caregiver Quality of Life Index Cancer
CRS – Caregiver Reaction Scale
CSI – Caregiver Strain Index
CSQ8 – Client Satisfaction Questionnaire
DCI – Dyadic-Coping-Inventory
ENRICH – ENRICH Marital Inventory
FACESII – Family Adaptability and Cohesion Evaluation Scale – II
FACIT-Sp – Functional Assessment of Chronic Illness Therapy-Spiritual Well-Being Scale
FACT – Functional Assessment of Cancer Therapy
FACT-G – Functional Assessment of Cancer Therapy - General
FACT-SWB – Functional Assessment of Cancer Therapy – Spiritual Well-Being Scale
FCI – Family Care Inventory

FES – Family Environment Scale
FFMQ-SF – Five Facet Mindfulness Questionnaire
HADS – Hospital Anxiety and Depression Scale
IES – Impact of Events Scale
LASA – Linear Analog Self-Assessment items
LCSS – Lewis Cancer Self-Efficacy Scale
LMSS – Lewis Mutuality and Sensitivity Scale
MBCB – Montgomery-Borgatta Caregiver Burden Scale
MOCS – Measure of Current Status
MSI – Marital Satisfaction Inventory
MSIS – Miller Social Intimacy Scale
MUIS – Mishel Uncertainty in Illness Scale
NCCNDT – National Comprehensive Cancer Network Distress Thermometer
PG13 – Prigerson Inventory of Complicated Grief-Short Form
POMB – Profile of Mood States-B
PROMIS SF Anxiety – 6-Item Patient Reported Outcomes Measurement Information System Short-Form Anxiety Measure
PROMIS SF Depression – 6-Item Patient Reported Outcomes Measurement Information System Short-Form Depression Measure
PROMIS SF Emotional Support – 4-Item Patient Reported Outcomes Measurement Information System Short-Form Emotional Support Measure
PROMIS SF Fatigue – 6-Item Patient Reported Outcomes Measurement Information System Short-Form Fatigue Measure
PTGI – Posttraumatic Growth Inventory
RDAS – Revised Dyadic Adjustment Scale
SA – Social Anxiety Scale
SC92 – Symptom Checklist – 92
SDS – Self-Rating Depression Scale
SF36 – 36-Item Short-Form Health Survey
SPSI – Social Problem Solving Inventory
SSQ – Social Support Questionnaire
SSS – Standard Self-Efficacy Scale
SV-POMS – Shortened Version Profile of Mood States

Supplementary Table 3. Study Characteristics, of Interventions Directed at Patients and Their Families

Trial (Country)	Study Design	Patient Population and Caregiver's Relationship to Patients	Caregiver Outcomes Assessed (Instrument)	Intervention		Comparator		Results
				Description	n	Description	n	
**Addington-Hall *et al*, 1992 (United Kingdom)** [82]	Multi-site, cluster-randomized controlled trial	Patients: terminal cancer. Setting = outpatient. Percentage female = 54%<br><br>Caregivers: family. Percentage female = 70%	Anxiety (HADS), anxiety (HADS)	Nurse coordinators, to coordinate services for patient and family	104	Usual care	99	Anxiety, before death: OR=0.65 (95% CI: 0.31-1.37)<br><br>Anxiety, 8 weeks after death: OR=0.51 (95% CI: 0.19-1.31)<br><br>Depression, before death: OR=0.77 (95% CI: 0.36-1.64)<br><br>Depression, 8 weeks after death: OR=0.63 (95% CI: 0.27-1.47)
**Clark *et al*, 2006 (USA)** [83]	Single-site, individually-randomized controlled trial	Patients: advanced cancer. Setting = outpatient<br><br>Caregivers: primary caregiver. Mean age = 59.5 years. Percentage female = 75%	Burden (interview), quality of life (LASA)	Eight 90-minute structured patient-focused multidisciplinary intervention focused on strategies to improve patients' quality of life	43	Standard medical care	40	Burden, at 4 weeks: 76.9 vs 76.2 (p=0.81)<br><br>Burden, at 8 weeks: 75.1 vs 75.8 (p=0.80)<br><br>Burden, at 27 weeks: 75.1 vs 77.2 (p=0.55)<br><br>Quality of life, at 4 weeks: 77.4 vs 75.9 (p=0.68)<br><br>Quality of life, at 8 weeks: 77.6 vs 76.3 (p=0.75)<br><br>Quality of life, at 27 weeks: 72.2 vs 78.9 (p=0.10)
**El-Jawahri *et al*, 2016 (USA)** [84]	Single-site, individually-randomized controlled trial	Patients: hematopoietic stem cell transplantation. Setting = inpatient. Mean age = 60. Percentage female = 57%<br><br>Caregiver: relative/friend who lived with patient or had in-person contact at least twice per week. Mean age = 54 years. Percentage female = 70%	Mood (HADS, PHQ-9), quality of life (CareGiver Oncology QOL questionnaire)	Inpatient palliative care focused on management of physical and psychological symptoms	49	Standard transplant care	45	Anxiety, at 2 weeks: 6.24 vs 6.55 (p=0.95)<br><br>Coping, at 2 weeks: 12.70 vs 11.59 (p=0.02)<br><br>Depression (HADS), at 2 weeks: 4.05 vs 6.00 (p=0.03)<br><br>Depression (PHQ-9), at 2 weeks: 4.51 vs 4.96 (p=0.99)<br><br>Quality of life, at 2 weeks: 118.72 vs 113.32 (p=0.24)

Trial (Country)	Study Design	Patient Population and Caregiver's Relationship to Patients	Caregiver Outcomes Assessed (Instrument)	Intervention		Comparator		Results
				Description	n	Description	n	
**El-Jawahri *et al*, 2017 (USA)** [85]	Single-site, individually-randomized controlled trial	Patients: incurable lung or noncolorectal gastrointestinal cancer. Setting = outpatient<br><br>Caregivers: caregiver identified by patient. Mean age = 57.4 years. Percentage female = 69%	Anxiety (HADS), mood (HADS), quality of life (SF36)	Early palliative care integrated with oncology care	137	Usual oncology care	138	Anxiety and mood, at 12 weeks: d = 0.30 (p=0.029)<br><br>Anxiety and mood, at 24 weeks: d = 0.15 (p=0.279)<br><br>Anxiety and mood, at 3 months before death: 6.84 vs 12.93 (p=0.002)<br><br>Anxiety and mood, at 6 months before death: 7.88 vs 11.60 (p=0.003)<br><br>Quality of life, physical component, at 12 weeks: d = 0.18 (p=0.183)<br><br>Quality of life, physical component, at 24 weeks: d=0.06 (p=0.669)<br><br>Quality of life, physical component, at 3 months before death: 52.69 vs 52.17 (p=0.664)<br><br>Quality of life, physical component, at 6 months before death: 53.27 vs 52.14 (p=0.299)<br><br>Quality of life, mental component, at 12 weeks: d = 0.12 (p=0.376)<br><br>Quality of life, mental component, at 24 weeks: d=0.06 (p=0.679)<br><br>Quality of life, mental component, at 3 months before death: 46.75 vs 46.01 (p=0.579)<br><br>Quality of life, mental component, at 6 months before death: 47.10 vs 45.91 (p=0.338)
**Kane *et al*, 1985 (USA)** [86]	Single-site, individually-randomized controlled trial	Patients: terminal cancer. Setting = home<br><br>Caregivers: significant other. Percentage female = 83%	Anxiety (GWM), depression (CESD), satisfaction	Hospice program	136	Usual care	110	Anxiety: p<0.05 for 3 of 5 cohorts (those completing 3/7, 4/7 and 5/7 interviews)<br><br>Depression: p>0.05

Trial (Country)	Study Design	Patient Population and Caregiver's Relationship to Patients	Caregiver Outcomes Assessed (Instrument)	Intervention		Comparator		Results
				Description	n	Description	n	
			(adapted scales)					Satisfaction with involvement in care: *p*<0.01 for 2 of 5 cohorts (those completing 5/7 and 6/7 interviews)
**Kissane *et al*, 2006 (Australia)** [87]<br><br>**Kissane *et al*, 2007 (Australia)** [88]	Multi-site, individually-randomized controlled trial	Patients: cancer prognosis of 6 months. Setting = outpatient. Mean age = 57 years. Percentage female = 49%<br><br>Caregivers: family member. Mean age = 37 years. Percentage female = 52%	Depression (BDI), distress (BSI), family functioning (FES, FAD), grief (BPQ), social adjustment (SAS)	Four to eight 90-minute family focused grief therapy sessions	233	Standard palliative care by home-care programs	130	Depression, at 6 months: Δ=0.64 (*p*=0.22)<br><br>Depression, at 13 months: Δ=0.26 (*p*=0.52)<br><br>Distress, at 6 months: Δ=0.04 (*p*=0.36)<br><br>Distress, at 13 months: Δ=0.11 (*p*=0.02)<br><br>Family functioning (FES), at 6 months: Δ=-0.22 (*p*=0.76)<br><br>Family functioning (FES), at 13 months: Δ=-0.07 (*p*=0.92)<br><br>Family functioning (FAD), at 6 months: Δ=0.06 (*p*=0.65)<br><br>Family functioning (FAD), at 13 months: Δ=0.03 (*p*=0.95)<br><br>Grief, at 13 months: Δ=-0.48 (*p*=0.92)<br><br>Social adjustment, at 6 months: Δ=0.01 (*p*=0.32)<br><br>Social adjustment, at 13 months: Δ=0.03 (*p*=0.43)
**Kissane *et al*, 2016 (USA)** [89]	Single-site, individually-randomized controlled trial	Patients: advanced cancer. Setting = mixed<br><br>Caregivers: partners and other relatives	Depression (BDI), grief (CGI)	6 family focused grief therapy sessions<br><br>10 family focused grief therapy sessions	59<br><br>56	Usual care	55	Depression: χ^2=1.00 (*p*=0.61)<br><br>Grief: χ^2=6.88 (*p*=0.03)
**Kleijn *et al*, 2021 (Netherlands)** [90]	Single-site, individually-randomized controlled trial	Patients: incurably ill cancer. Setting = mixed<br><br>Caregivers: informal caregiver. Mean age =	Anxiety (HADS), burden (CRA), depression	Four weekly 1-hour life review therapy with memory specificity	35	Usual care	29	Anxiety, over time: F=1.27 (*p*=0.29)<br><br>Burden, disrupted schedule, over time: F=0.07 (*p*=0.93)

Trial (Country)	Study Design	Patient Population and Caregiver's Relationship to Patients	Caregiver Outcomes Assessed (Instrument)	Intervention		Comparator		Results
				**Description**	**n**	**Description**	**n**	
		61.6 years. Percentage female = 61%	(HADS), distress (HADS), posttraumatic growth (PTGI)	training sessions for patients, led by psychologist				Burden, financial problems, over time: F=0.33 (*p*=0.72)<br><br>Burden, lack of family support, over time: F=0.31 (*p*=0.74)<br><br>Burden, health problems, over time: F=0.23 (*p*=0.80)<br><br>Burden, self-esteem, over time: F=4.57 (*p*=0.01)<br><br>Depression, over time: F=0.92 (*p*=0.40)<br><br>Distress, over time: F=1.27 (*p*=0.29)<br><br>Posttraumatic growth, total, over time: F=0.62 (*p*=0.54)<br><br>Posttraumatic growth, relating to others, over time: F=0.31 (*p*=0.73)<br><br>Posttraumatic growth, new possibilities, over time: F=0.85 (*p*=0.43)<br><br>Posttraumatic growth, personal strength, over time: F=1.38 (*p*=0.26)<br><br>Posttraumatic growth, spiritual change, over time: F=0.29 (*p*=0.75)<br><br>Posttraumatic growth, appreciation of life, over time: F=0.77 (*p*=0.47)
**McDonald *et al*, 2017 (Canada)** [91]	Multi-site, cluster-randomized controlled trial	Patients: stage III-IV cancer. Setting = outpatient<br><br>Caregivers: not defined. Mean age = 57.5 years. Percentage female = 65%	Satisfaction with care (FAMCARE), quality of life (CQOLC, SF36)	Early outpatient palliative care	94	Standard oncology care	88	Satisfaction with care, at 3 months: β=1.47 (*p*<0.01)<br><br>Satisfaction with care, at 4 months: β=0.89 (*p*=0.02)<br><br>Quality of life (CQOLC), at 3 months: β=0.11 (*p*=0.92)

Trial (Country)	Study Design	Patient Population and Caregiver's Relationship to Patients	Caregiver Outcomes Assessed (Instrument)	Intervention		Comparator		Results
				Description	n	Description	n	
								Quality of life (CQOLC), at 4 months: β=0.50 (*p*=0.51)<br><br>Quality of life (SF36), physical component, at 3 months: β=0.07 (*p*=0.83)<br><br>Quality of life (SF36), physical component, at 4 months: β=0.17 (*p*=0.20)<br><br>Quality of life (SF36), mental component, at 3 months: β=0.07 (*p*=0.87)<br><br>Quality of life (SF36), mental component, at 4 months: β=-0.17 (*p*=0.60)
**O'Hara *et al*, 2010 (USA)** [92]	Single-site, individually-randomized controlled trial	Patients: advanced cancer. Setting = outpatient<br><br>Caregivers: someone close to the patient who was involved with care. Mean age = 58.9 years. Percentage female = 77%	Burden (Montgomery Borgatta Caregiver Burden Scale)	Educate, Nurture, Advise Before Life Ends (ENABLE) intervention: four weekly telephone sessions with patients by specialized nurse educators, and monthly calls thereafter	108	Standard care	90	Burden: $p > 0.05$
**Ringdal *et al*, 2001 (Norway)** [93]	Single-site, cluster-randomized controlled trial	Patients: advanced cancer. Setting = mixed. Mean age = 68.4 years. Percentage female = 47%<br><br>Caregivers: family or friend. Mean age = 56.3 years. Percentage female = 68%	Grief (TRIG)	Team-based comprehensive palliative care	113	Conventional care	70	Grief, 0 to 13 months: f=8.15 (*p*<0.01)
**Schenker *et al*, 2018 (USA)** [94]	Single-site, individually-	Patients: locally advanced or metastatic pancreatic cancer.	Burden (Zarit burden interview),	In-person palliative care visits with	20	Usual care	10	Burden, at 3 months: Δ=3.0 vs Δ=-0.3<br><br>Mood, anxiety, at 3 months: Δ=-2.1 vs Δ=-2.3

Trial (Country)	Study Design	Patient Population and Caregiver's Relationship to Patients	Caregiver Outcomes Assessed (Instrument)	Intervention		Comparator		Results
				**Description**	**n**	**Description**	**n**	
	randomized controlled trial	Setting = outpatient. Mean age = 63. Percentage female = 50%<br><br>Caregivers: family member or friend. Mean age = 62. Percentage female = 53%	mood (HADS)	speciality-trained palliative care physician				Mood, depression, at 3 months: Δ=-1.5 vs -1.3<br><br>Mood, distress, at 3 months: Δ=-0.9 vs -1.6
**Steel *et al*, 2016 (USA)** [95]	Single-site, individually-randomized controlled trial	Patients: hepatocellular carcinoma, cholangiocarcinoma, gallbladder carcinoma, neuroendocrine carcinoma, pancreatic carcinoma or other primary cancers that had metastasized to liver. Setting = mixed. Mean age = 61 years. Percentage female = 27%<br><br>Caregivers: family caregiver	Depression (CESD), stress	Patient access to a psychoeducation website and face-to-face and phone contact contact with a care coordinator experienced in cognitive behavioral therapy and psycho-oncology	124	Enhanced usual care: usual medical care with contact by a care coordinator and appropriate education and referral if patients or caregivers scored high on depression or pain measures	100	Depression, at 6 months: d=0.37 (*p*=0.10)<br><br>Stress, at 6 months: d=0.75 (*p*=0.05)
**Xu et al, 2021 (China)** [96]	Single-site, individually-randomized controlled trial	Patients: terminal cancer. Setting = inpatient. Mean age = 74.8 years. Percentage female = 47%<br><br>Caregivers: immediate family member. Mean age = 37.0 years. Percentage female = 57%	Anxiety (HAMA), depression (HAMD), satisfaction (NSS), sleep quality (PSQI), stress (RSS)	Palliative care intervention encompassing face-to-face communication, death education, psychological support, and symptom-based conservative treatment	51	Routine end-of-life care	51	Anxiety, baseline to after intervention: Δ=-4.12 vs Δ=-1.40 (*p*<0.01)<br><br>Depression, baseline to after intervention: Δ=-2.55 vs Δ=-0.88 (*p*<0.01)<br><br>Satisfaction: 96% vs 82% (*p*<0.05)<br><br>Sleep, baseline to after intervention: Δ=-3.98 vs Δ=-1.43 (*p*<0.01)<br><br>Stress, psychological stress, baseline to after intervention: Δ=-1.46 vs Δ=-0.80 (*p*<0.05)

Trial (Country)	Study Design	Patient Population and Caregiver's Relationship to Patients	Caregiver Outcomes Assessed (Instrument)	Intervention		Comparator		Results
				Description	n	Description	n	
								Stress, disrupted life, baseline to after intervention: Δ=-1.39 vs Δ=-0.65 (p<0.01)<br><br>Stress, negative emotions, baseline to after intervention: Δ=-1.18 vs Δ=-0.59 (p<0.01)

Legend:
BDI – Beck Depression Inventory
BPQ – Bereavement Phenomenlogy Questionnaire
BSI – Beck Symptom Inventory
CESD – Center for Epidemiological Studies Depression Scale
CGI – Complicated Grief Inventory
CQOLC – Caregiver Quality of Life Index – Cancer
CRA – Caregivers Reaction Assessment Scale
FAD – Family Assessment Device
FAMCARE – Family Satisfaction with End of Life Care Questionnaire
FES – Family Environment Scale
GWM – General Well-Being Measure
HADS – Hospital Anxiety and Depression Scale
HAMA – Hamilton Anxiety Scale
HAMD – Hamilton Depression Scale
LASA – Linear Analog Self-Assessment items
NSS – Nursing Satisfaction Survey Scale
PHQ-9 – Patient Health Questionnaire-9
PSQI – Pittsburgh Sleep Quality Index
PTGI – Posttraumatic Growth Inventory
RSS – Relative Stress Scale
SAS – Social Adjustment Scale
SF36 – 36-Item Short-Form Health Survey
TRIG – Texas Revised Inventory of Grief

Supplementary Figure 1

1.1

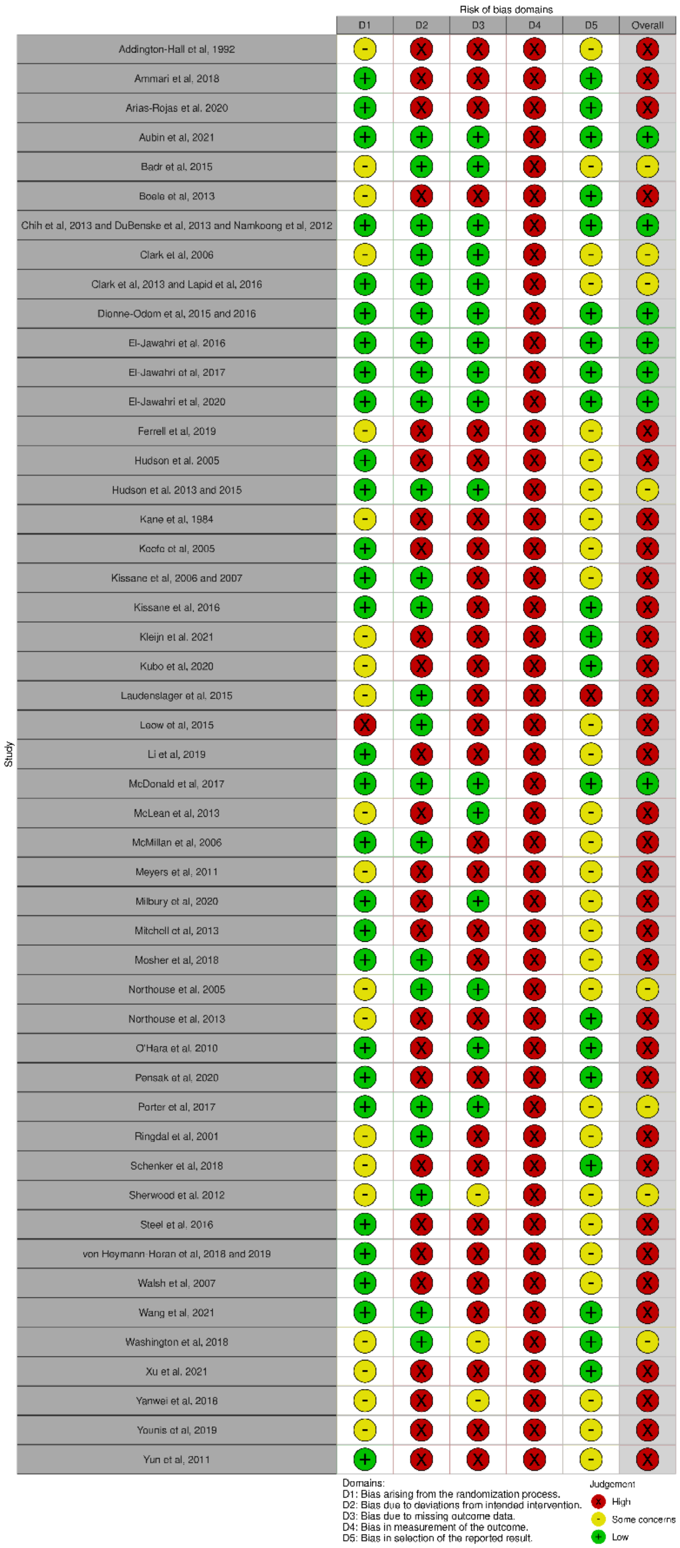

Legend:
Supplementary Figure 1.1. Cochrane Risk of Bias Assessment By Study.

1.2

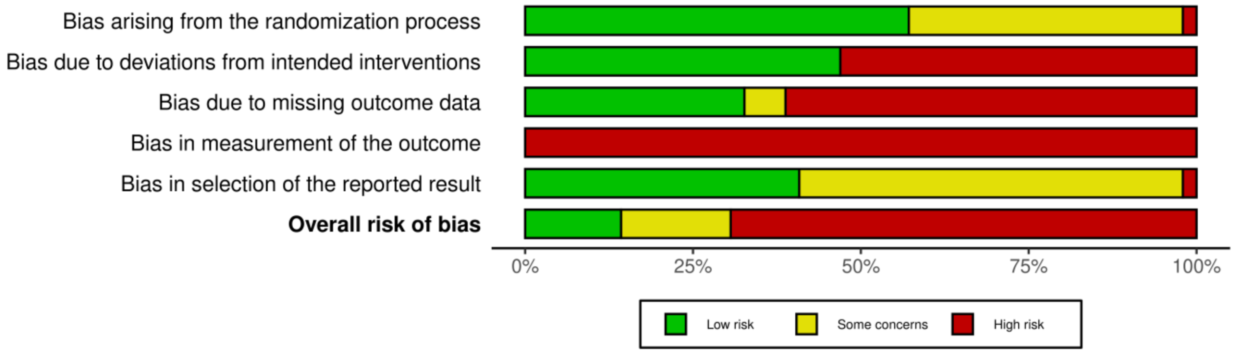

Legend:
Supplementary Figure 1.2. Cochrane Risk of Bias Assessment Summary.

Supplementary Figure 2

2.1

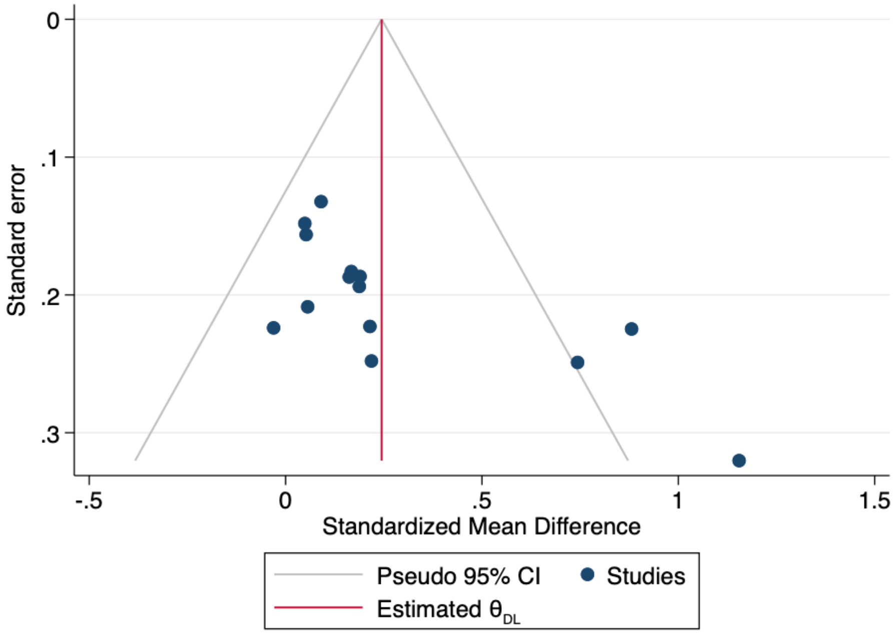

Legend:
Supplementary Figure 2.1. Overall Quality of Life Funnel Plot, for Assessment of Publication Bias at 1-3 Months (*p*<0.001).

2.2

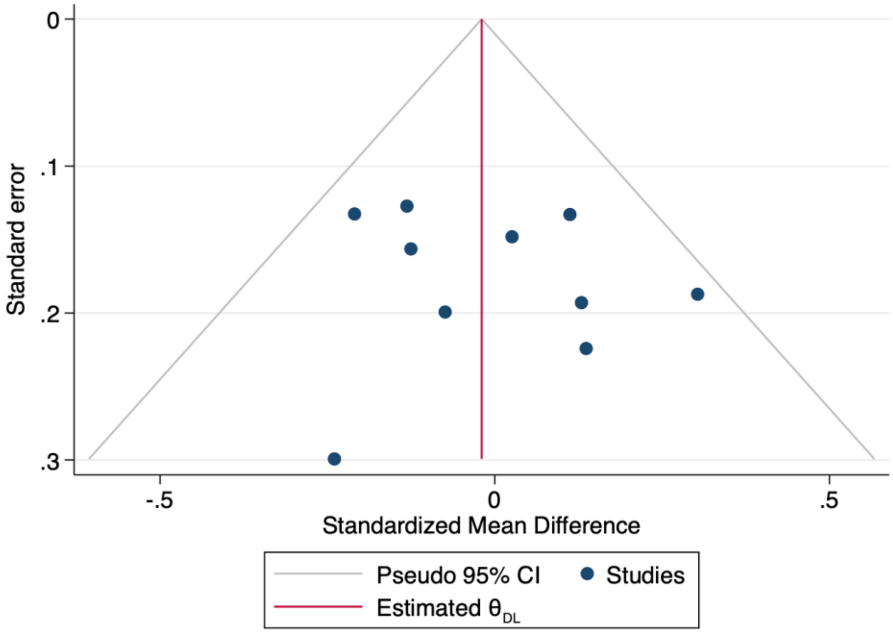

Legend:
Supplementary Figure 2.2. Physical Well-being Funnel Plot, for Assessment of Publication Bias at 1-3 Months (*p*=0.533).

2.3

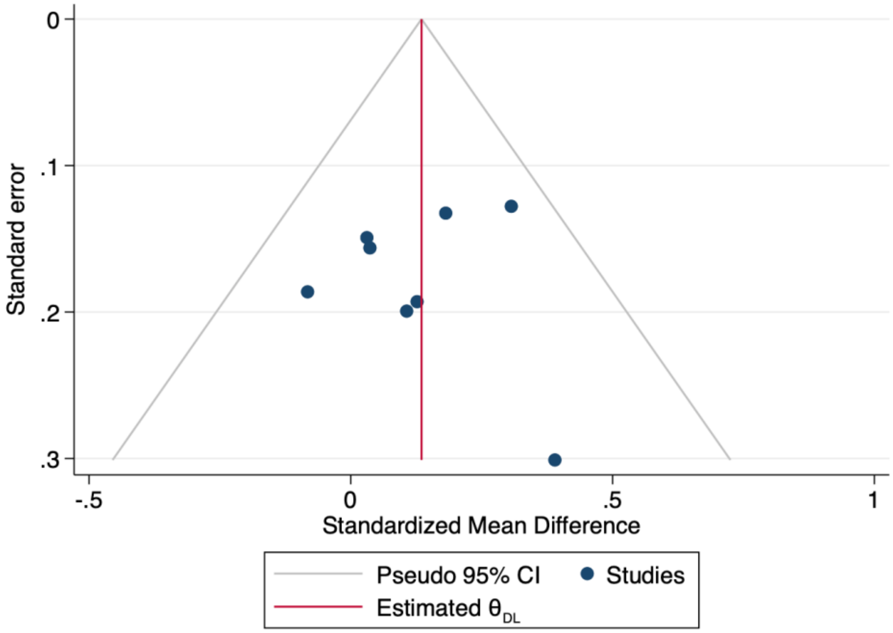

Legend:
Supplementary Figure 2.3. Mental Well-being Funnel Plot, for Assessment of Publication Bias at 1-3 Months (*p*=0.819).

2.4

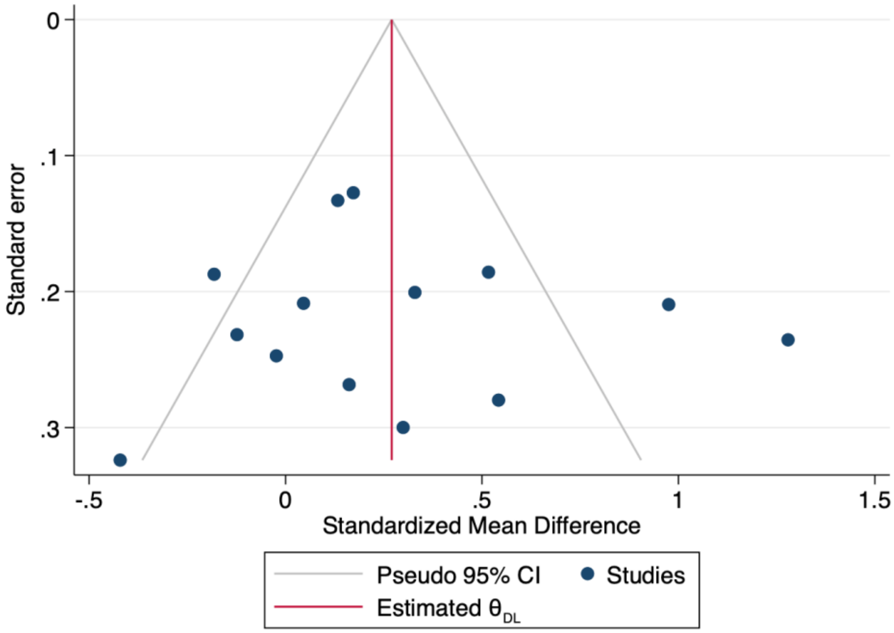

Legend:
Supplementary Figure 2.4. Anxiety Funnel Plot, for Assessment of Publication Bias at 1-3 Months (*p*=0.868).

2.5

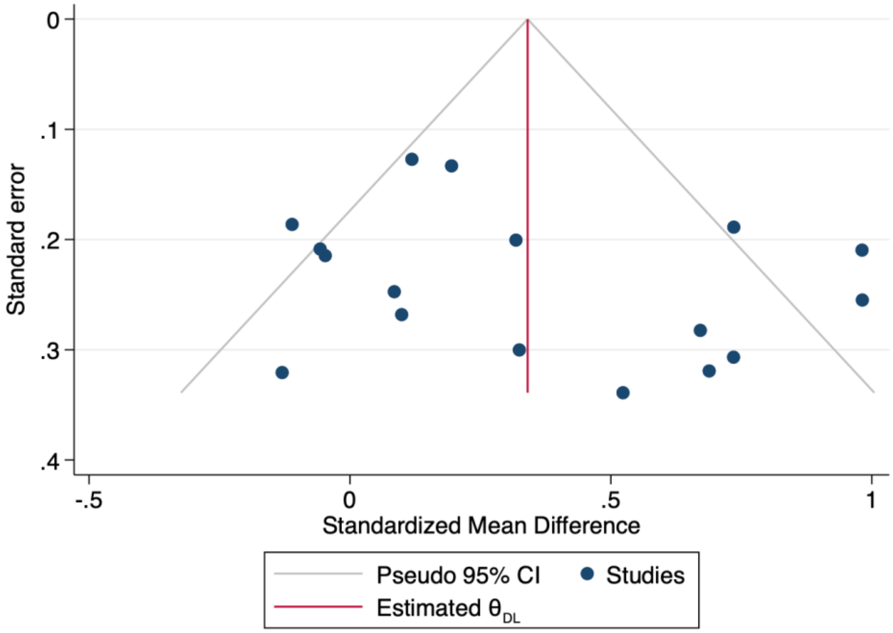

Legend:
Supplementary Figure 2.5. Depression Funnel Plot, for Assessment of Publication Bias at 1-3 Months (*p*=0.284).

Supplementary Figure 3

3.1

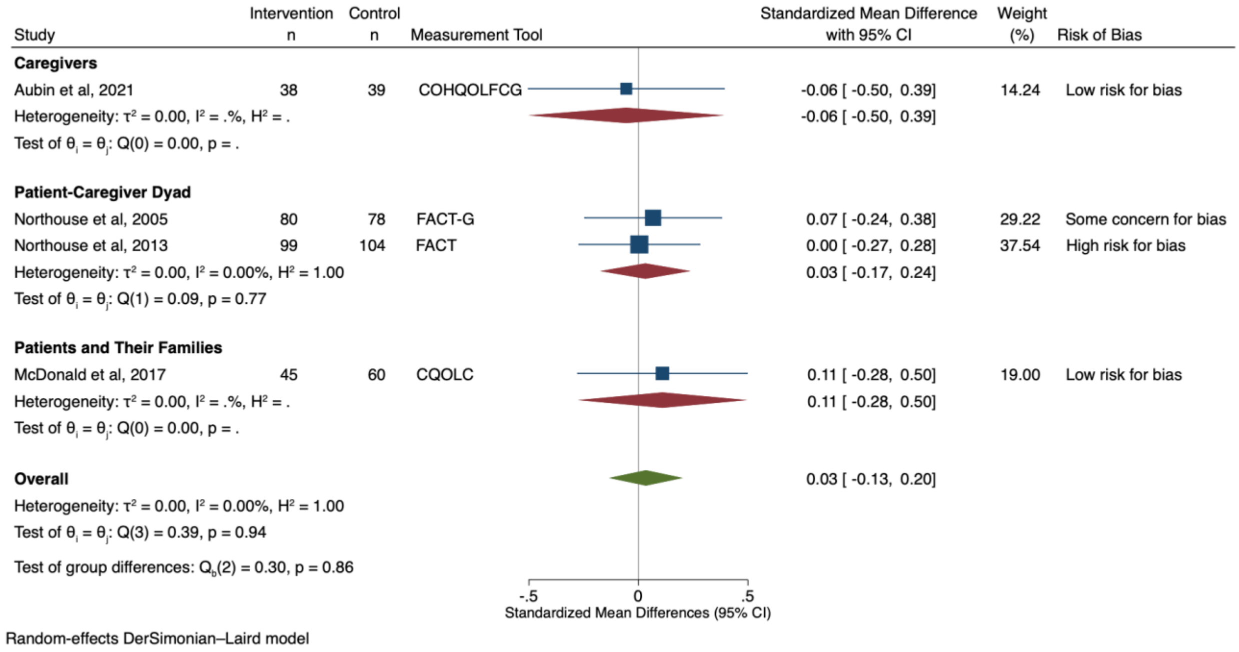

Legend:
Supplementary Figure 3.1. Overall Quality of Life Caregiver-Reported Outcomes, at 4-6 Months.

COHQOLFCG – City of Hope Quality of Life Family Caregiver Version
CQOLC – Caregiver Quality of Life Index – Cancer
FACT – Functional Assessment of Cancer Therapy
FACT-G – Functional Assessment of Cancer Therapy - General

3.2

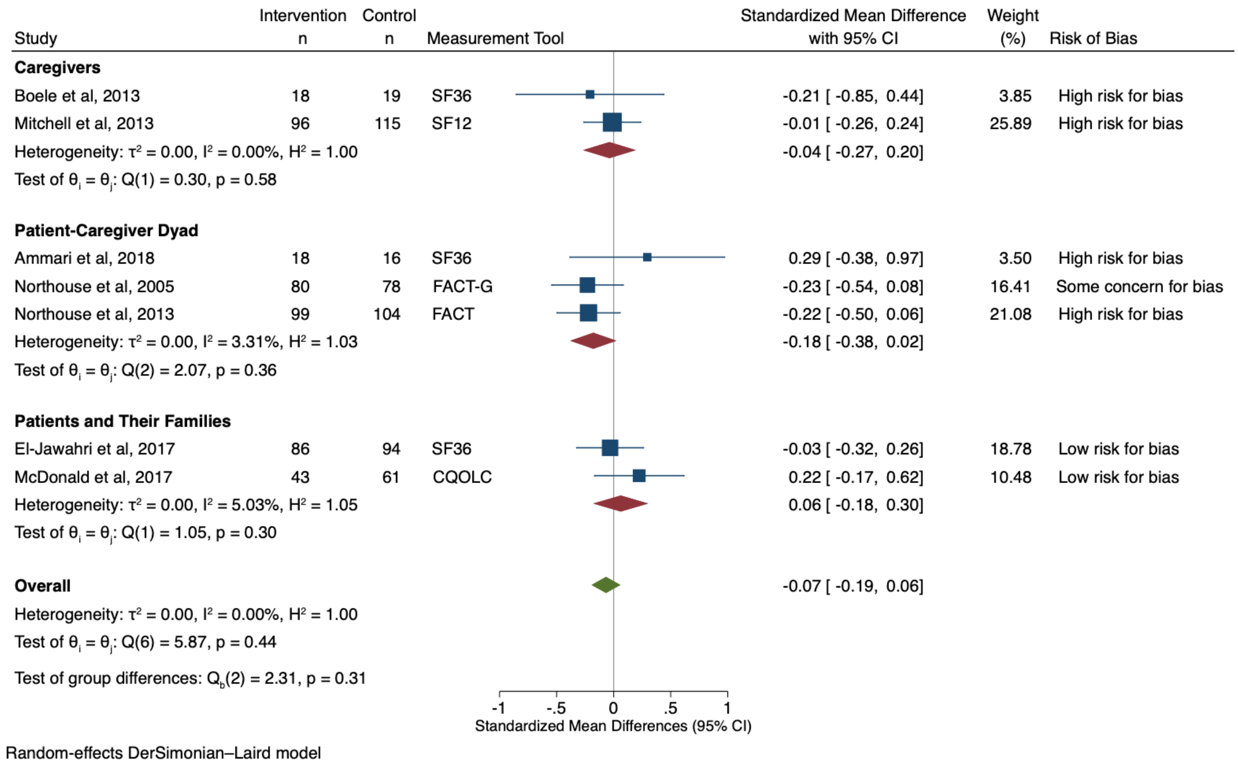

Legend:
Supplementary Figure 3.2. Physical Well-Being Caregiver-Reported Outcomes, at 4-6 Months.

CQOLC – Caregiver Quality of Life Index – Cancer
FACT – Functional Assessment of Cancer Therapy
FACT-G – Functional Assessment of Cancer Therapy - General
SF12 – 12-Item Short-Form Health Survey
SF36 – 36-Item Short-Form Health Survey

3.3

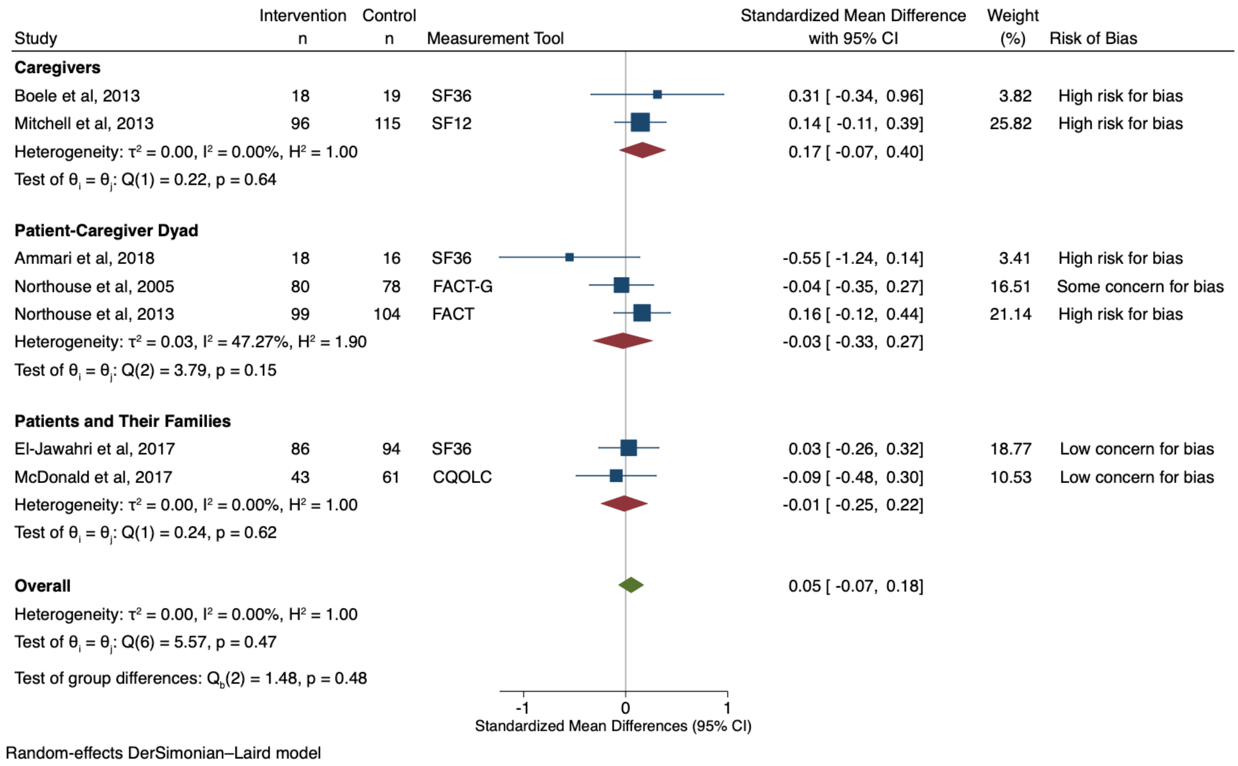

Legend:
Supplementary Figure 3.3. Mental Well-Being Caregiver-Reported Outcomes, at 4-6 Months.

CQOLC – Caregiver Quality of Life Index – Cancer
FACT – Functional Assessment of Cancer Therapy
FACT-G – Functional Assessment of Cancer Therapy - General
SF12 – 12-Item Short-Form Health Survey
SF36 – 36-Item Short-Form Health Survey

3.4

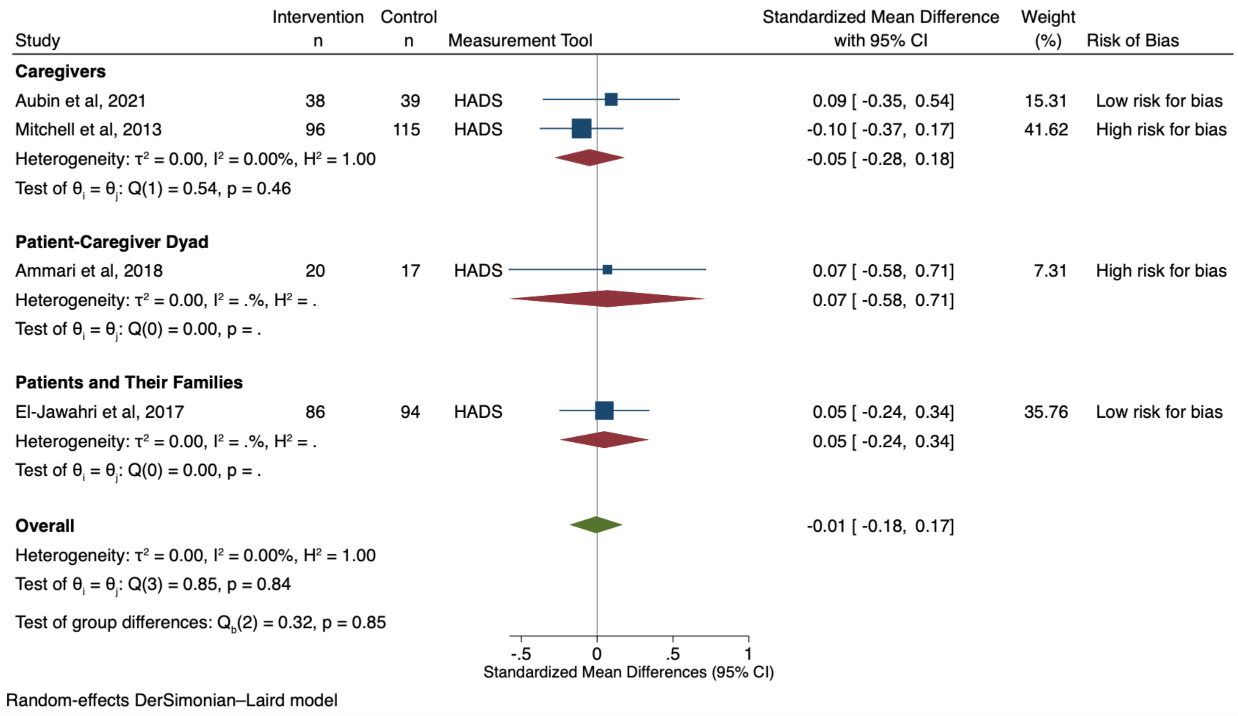

Legend:
Supplementary Figure 3.4. Anxiety Caregiver-Reported Outcomes, at 4-6 Months.

HADS – Hospital Anxiety and Depression ScaleHAMA – Hamilton Anxiety Scale

3.5

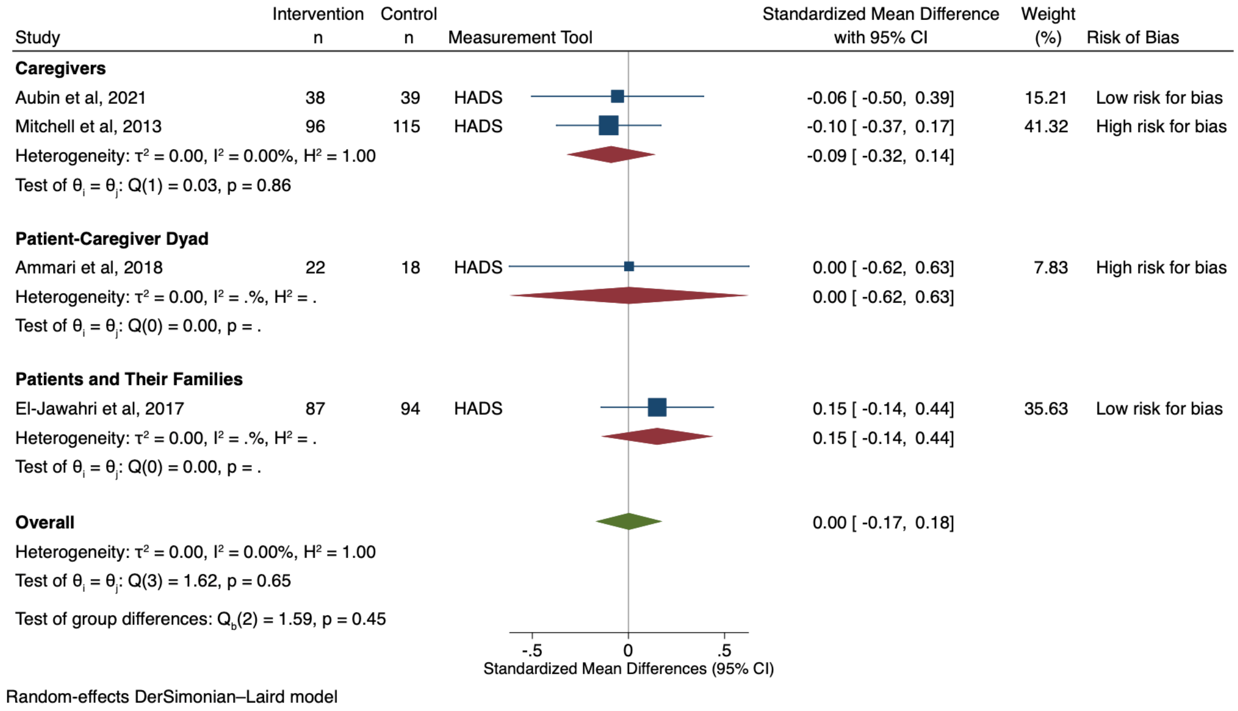

Legend:
Supplementary Figure 3.5. Depression Caregiver-Reported Outcomes, at 4-6 Months.

HADS – Hospital Anxiety and Depression Scale
